# Good practices and recommendations for using and benchmarking computational metabolomics metabolite annotation tools

**DOI:** 10.1007/s11306-022-01963-y

**Published:** 2022-12-05

**Authors:** Niek F. de Jonge, Kevin Mildau, David Meijer, Joris J. R. Louwen, Christoph Bueschl, Florian Huber, Justin J. J. van der Hooft

**Affiliations:** 1 Bioinformatics Group, Wageningen University, Wageningen, the Netherlands; 2 Department of Analytical Chemistry, Biochemical Network Analysis Lab, University of Vienna, Vienna, Austria; 3 Centre for Digitalization and Digitality (ZDD), University of Applied Sciences Düsseldorf, Düsseldorf, Germany; 4 Department of Biochemistry, University of Johannesburg, Johannesburg, South Africa

**Keywords:** Untargeted metabolomics, Mass spectrometry, Mass fragmentation spectra, Metabolite annotation and identification, Machine learning, Benchmarking

## Abstract

**Background:**

Untargeted metabolomics approaches based on mass spectrometry obtain comprehensive profiles of complex biological samples. However, on average only 10% of the molecules can be annotated. This low annotation rate hampers biochemical interpretation and effective comparison of metabolomics studies. Furthermore, de novo structural characterization of mass spectral data remains a complicated and time-intensive process. Recently, the field of computational metabolomics has gained traction and novel methods have started to enable large-scale and reliable metabolite annotation. Molecular networking and machine learning-based *in-silico* annotation tools have been shown to greatly assist metabolite characterization in diverse fields such as clinical metabolomics and natural product discovery.

**Aim of review:**

We highlight recent advances in computational metabolite annotation workflows with a special focus on their evaluation and comparison with other tools. Whilst the progress is substantial and promising, we also argue that inconsistencies in benchmarking different tools hamper users from selecting the most appropriate and promising method for their research. We summarize benchmarking strategies of the different tools and outline several recommendations for benchmarking and comparing novel tools.

**Key scientific concepts of review:**

This review focuses on recent advances in mass spectral library-based and machine learning-supported metabolite annotation workflows. We discuss large-scale library matching and analogue search, the current bloom of mass spectral similarity scores, and how molecular networking has changed the field. In addition, the potentials and challenges of machine learning-supported metabolite annotation workflows are highlighted. Overall, recent developments in computational metabolomics have started to fundamentally change metabolomics workflows, and we expect that as a community we will be able to overcome current method performance ambiguities and annotation bottlenecks.

## Background & motivation

Metabolites are key functional parts of biology with roles in metabolism, nutrition, intra- and inter-organism species communication, and signalling pathways. In general, they are important contributors to an organism’s growth and health (Fiehn, 2002). A growing number of metabolites has been discovered in recent years; hence, we increasingly appreciate the large chemical space that nature can produce and use (e.g., Wishart et al. (2022)). Reusing this large, currently mostly unexplored chemical space for our needs is promising, such as for the development of therapeutics or the finding of biomarkers for early detection of disease or various risks. To measure and understand this chemical space, untargeted metabolomics approaches have gained traction over the last two decades, fuelled by technical advances in analytical equipment as well as computational advances that support *in-silico* structural annotation of the generated information-dense metabolomics profiles (e.g., Misra (2021)). In this respect, the ideal experimental analysis of a sample in any untargeted metabolomics approach would report the structural identities (i.e., the chemical name and structure) of all metabolites and their absolute abundances. Unfortunately, today, this is still far from reality and will likely not be achieved any time soon. In the real world, techniques available for metabolite annotation are nuclear magnetic resonance (NMR) spectroscopy and mass spectrometry (MS) coupled to either gas chromatography (GC) or liquid chromatography (LC). Currently, LC-high-resolution MS (LC-HRMS) is the favoured analytical technique for untargeted analytical measurements with the aim of identifying and characterising many of the samples’ constituents. LC-HRMS is versatile, can easily be customised to researchers’ needs, and is extraordinarily sensitive. However, apart from the measurements themselves, the raw data analysis for unravelling the metabolites’ identities is cumbersome and error-prone (Alseekh et al., 2021), especially when no authentic reference standards are available for identity confirmation (Metabolomics Standards Initiative [MSI] Metabolite Identification [MI] level 1 identification (Members of the Metabolomics Standards Initiative et al., 2007; Sumner et al., 2007)).

Limited levels of metabolite annotation and low availability of chemical reference standards are widely recognized as a severe bottleneck for the biological interpretation of many research activities (Beniddir et al., 2021; da Silva et al., 2015; Dunn et al., 2012; Peisl et al., 2018; Stein, 2012; Tsugawa, 2018). This shortcoming could partly be addressed with great financial and manual labour efforts (i.e., production of many authentic reference standards), but restrictions due to high costs and/or limitations in available quantities make large improvements in this area unlikely. A less expensive and more versatile solution comes with *in-silico* approaches, which harness computing workflows from advanced machine learning and statistical approaches to predict the relevance and structural properties of chemical entities measured by the mass spectrometer with sufficient accuracy. Here, mass fragmentation spectra (MS/MS spectra) acquired through data dependent acquisition (DDA) or data independent acquisition (DIA) alternatives have demonstrated their merits in adding structural information to metabolomics profiles, as we will also demonstrate throughout this review. As such, computational metabolomics tools that capitalize on MS and MS/MS information are a pragmatic solution since it is unlikely that we will ever cover the true chemical diversity in nature exhaustively with available reference standards given the vastness of estimated natural chemical space (Polishchuk et al., 2013; Shrivastava et al., 2021).

*In-silico* annotation methods are typically employed in combination with structure and spectral databases, from which the tools learn to recognize chemical structures from LC-HRMS/MS data or even predict chemical properties for MS/MS data acquired from novel molecules (Blaženović et al., 2017, 2018). The comparisons and predictions typically result in scores for the observed query MS/MS spectra and the respective database entries that can then be ranked accordingly. However, it is important to stress that without further experimental validation or available complementary structural information, the use of *in-silico* annotation approaches only leads to MSI MI level 2 or 3 annotations, but no definite identification of the molecular structures, even in cases with perfect scores. Nevertheless, these annotations are of utmost importance and serve as an excellent starting point for subsequent validation with newly acquired standards, organic synthesis approaches, or for prioritization strategies.

In principle, *in-silico* methods are suited to appreciate the true chemical diversity in natural extracts. However, *in-silico* annotation strategies suffer from low accuracies (i.e., a high number of false-positives) and often do not report the correct annotation as the top hit but rather within the first 5 or 10 hits. Most analytical (bio)chemists are not used to such low accuracies, and they are often tempted to simply use the best-scoring hit. However, this should be avoided as errors during this annotation will propagate to the biological interpretation. Thus, akin to monitoring and ensuring an adequate LC-HRMS performance during the analytical measurements, the predictions and performance of the *in-silico* methods should also be tested, and it should be verified whether they are correct or not. Ideally, the different available *in-silico* methods are compared and the best performing one for the analytical setup and research question at hand is subsequently used.

Metabolome mining approaches based on large-scale mass spectral comparisons and machine learning are becoming increasingly popular (Beniddir et al., 2021). Noteworthy and widely-adopted methods are molecular networking (Aron et al., 2020; Nothias et al., 2020; Wang et al., 2016) and, in general, methods that group molecules of likely high chemical similarity based on their MS/MS spectra, i.e., mass spectral networking. Here, identified (or annotated) molecules allow the propagation and use of this chemical identity to improve the annotation of other unidentified or unannotated members of this metabolite group or molecular family, also coined Network Annotation Propagation (da Silva et al., 2018).

The constant development of novel tools drives continuous increases in prediction power and thus reliability of these *in-silico* methods. In general, to benchmark such tools, analytical data of known molecules is processed and analysed, and the obtained results are compared to the known identity of the molecules under investigation. Then, figures of merit such as accuracy or false discovery rates are calculated. Unfortunately, the test datasets used are not standardized, which often makes it difficult to compare different tools. Consequently, the community would greatly benefit from standards and reused datasets among the different tools to allow for better comparison. Especially end-users of the tools such as analytical chemists and biologists would benefit as this would greatly increase their confidence in the tools and their results.

In this critical review, we first focus on mass spectral similarity metrics that compute similarity scores between MS/MS spectra in the context of mass spectral library matching, large-scale mass spectral comparisons, and mass spectral networking. Second, we review current tools making use of machine learning / deep learning in metabolite annotation and discuss the challenges in fair benchmarking (validating) and comparison of different *in-silico* metabolite annotation tools. Finally, we provide recommendations on how to discover the strengths and weaknesses of the tools. In addition, we highlight the methods we deem to be at the forefront of the current state of the art in metabolite annotation and stress their limitations as well as promising avenues for further research.​​

## Applications of mass spectral matching

MS/MS spectra obtained by LC–MS/MS analysis can be used to generate key insights from the wealth of data generated by high throughput metabolomics. Here, we will discuss two key uses they find in computational tools, namely i) mass spectral library matching for metabolite annotation including both direct matching and analogue search, and ii) in the organisation and exploration of the many MS/MS spectra generated in metabolomics profiles at once.

**Fig. 1:**
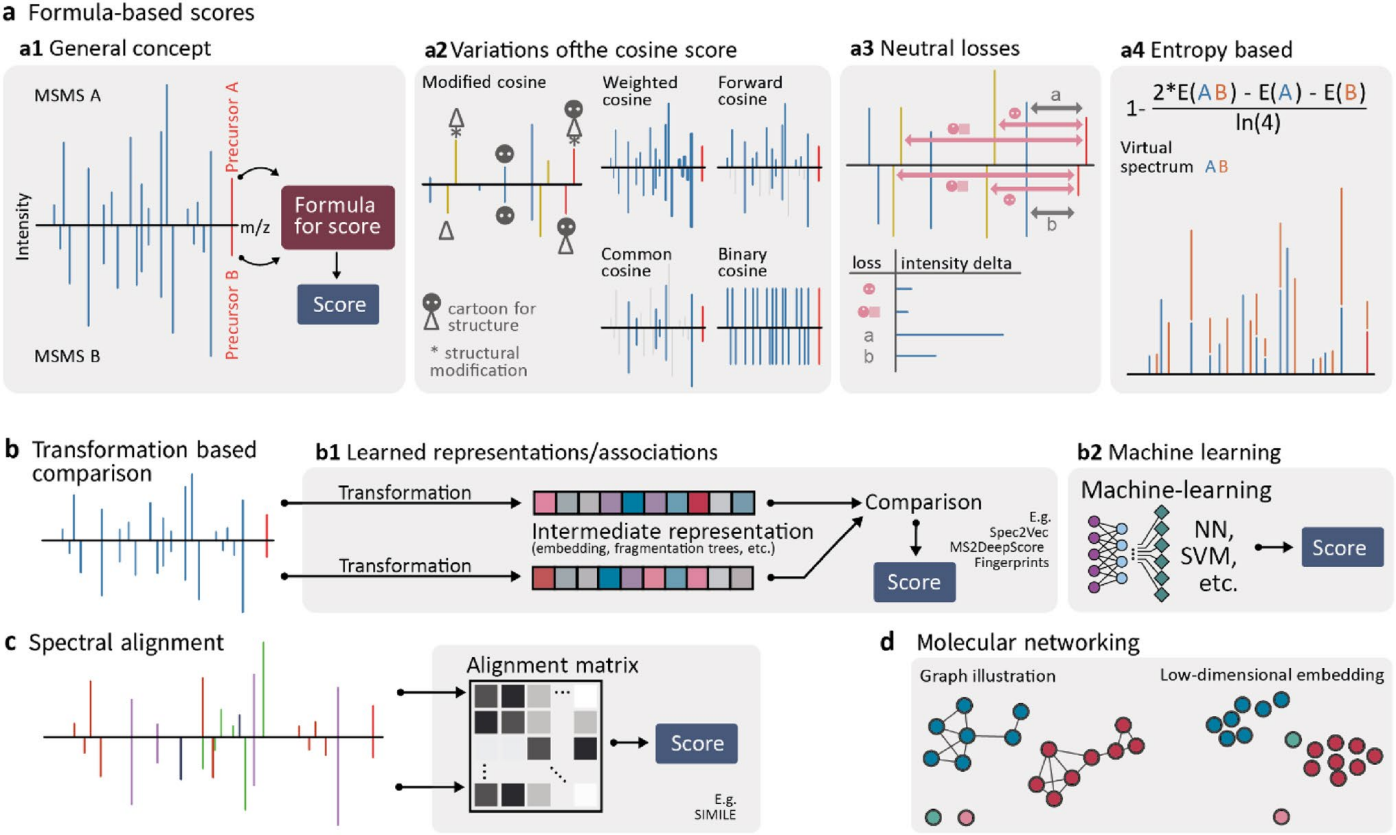
Overview of different spectral comparison (**a–c**) and spectral organisation methods (**d**) for two MS/MS spectra A and B. a1) Using mass spectral binning (i.e., to account for small *m/z* value differences), mass fragmentation spectra are transformed into vectors that are subsequently compared using mathematical formulas. a2) Modifications of the binning schema can account for other differences than *m/z* values (e.g., account for neutral losses, use only fragments present in both spectra, etc.). a3) Besides the actual mass fragment signals, neutral losses within or between spectra alone can serve as input for the spectral comparisons. a4) The Entropy score is a recently developed and high-performing metric for spectral comparisons. b1) Spectral comparison can be based on automatically computer-learned representations (i.e., alternatives to fragment spectral binning). b2) Comparison of MS/MS spectra can be achieved automatically with machine/deep learning methods and thus correlate better with structural similarity (NN: Neural Networks, SVM: Support Vector Machines). **c** Fragment spectra can be “aligned” similar to sequence alignment, which will report sub-spectra with overlapping fragments (i.e., certain structure parts of the two molecules, SIMILE: Significant Interrelation of MS/MS Ions via Laplacian Embedding). **d** Many MS/MS spectra can be organised into groups (molecular networking or mass spectral networking) or embedded in a lower subspace (a proxy for structural similarity)

Considering the first task, this can be subdivided into the identification of molecules using authentic reference standards (MSI MI level 1) and the general annotation with spectral databases (MSI MI level 2) (Blaženović et al., 2018; da Silva et al., 2018). To achieve metabolite identification, LC-HRMS/MS data of authentic standards and experimental samples are acquired with the same analytical settings thus leading to almost identical MS/MS spectra, retention times, MS1 adduct and isotope information. Hence, simple scoring methods and stringent cut-off values often suffice for matching and thus trusted identification. While level 1 identification is clearly the ideal aim, the unavailability of reference standards, as well as strong reference library bias towards [M + H] + or [M-H]- ions rather than a more complete adduct coverage, commonly causes  ~95% of measured spectra lacking respective molecule identifications (Blaženović et al., 2018; da Silva et al., 2015). To overcome this drawback, more flexible spectral matching approaches are used to match experimental MS/MS spectra to a broader set of reference MS/MS spectra from different analytical setups available in various reference databases. Here, mass spectral differences need to be tolerated to a much greater extent to query for plausible candidates. As commonly used scoring methods report a continuous value on the spectral similarity, they often fail to separate correct and incorrect matches leading to high numbers of incorrect annotations (Li et al., 2021; Scheubert et al., 2017). Thus, during mass spectral library matching, novel and improved scoring methods need to account for differences in mass spectral fragmentation patterns to allow correct matching spectra of identical molecules acquired on different machines, all while avoiding matching different molecules exhibiting similar mass fragmentation patterns (Fig. 1).

The second task of spectral matching is aimed toward the annotation of structurally related molecules as well as the organisation of vast amounts of mass spectral data into groups of molecules with high structural similarity. In the absence of a corresponding reference standard, structurally related molecules can serve as seeds for manual structural investigation. Moreover, even without in-depth manual structural analysis, it can be useful for biological and chemical interpretation to have an overview of structurally similar molecules (e.g., metabolites of the same chemical class or metabolic pathway). With respect to scoring, unlike classical database matching itself, this organisation requires structurally similar molecules to be recognized (Bero et al., 2017; Huber et al., 2021b). Consequently, such approaches require more sophisticated mass spectral matching methods, and ideally, the calculated score of two MS/MS spectra obtained from different molecules should reflect and correlate with the molecules’ structural similarity in a continuous fashion rather than confirming them to be identical or not. In such comparisons, even small structural differences (e.g., single or double-bonds, presence/absence of phosphorylation), can lead to quite dissimilar MS/MS spectra with *m/z* shifts for certain fragments as well as new fragments, in combination with altered relative intensity values. Respective scores ideally should be able to take such modifications and the resulting differences in the MS/MS spectra explicitly or implicitly into consideration. Furthermore, with respect to the organisation of mass spectral data, it has been observed that traditional scores (e.g., the classical cosine score) show suboptimal performance (Schollée et al., 2017). As a result, the community has started to develop more sophisticated approaches that automatically account for structural differences observed in the respective MS/MS spectra. In this section, we will discuss commonly used mass spectral similarity scores used for mass spectral annotation and organisation, and the impact of novel and recently proposed mass spectral similarity metrics.

### Library matching

Currently, metabolomics applications use diverse scoring approaches, ranging from measures based on spectra themselves to methods utilising machine learning predictions and mass spectral embeddings (a learned vector representation of mass spectra). The most used score is the cosine score (Fig. 1 a1). It converts two MS/MS spectra to two equally sized vectors through mass peak binning and establishes their dot-product. Numerous flavours of this score exist, differing primarily in which information of the fragmentation patterns are used for matching or are taken into consideration and how these are weighted (Fig. 1 a2). The different cosine scores have established themselves as standards in the metabolomics field. However, depending on the task and size of the dataset, their performance may not be optimal when compared to other scoring approaches (Huber et al., 2021a; Li et al., 2021), nor is it easy to set default thresholds optimally for all experiments (Scheubert et al., 2017). Hence, research into improving and developing better mass spectral matching scores and assessing their optimal use is still ongoing.

A recent contribution to the study of mass spectral similarity scores was published by Li and colleagues (Li et al., 2021) with the development of the Entropy score. This novel score measures the difference between two spectra as the difference in entropy between the individual spectra and a combined spectrum composed of the peaks of both spectra (Fig. 1 a4). The authors compared their method to 42 alternative similarity scoring approaches and demonstrated that their entropy score achieves the best performance in an evaluation of 25,555,973 pairwise spectral comparisons based on a large set of 434,287 MS/MS spectra representing 25,138 molecules from the NIST20 database. The authors varied score thresholds and measured corresponding false discovery rates. A particularly interesting aspect of their evaluation was the use of *in-silico* spectral noise to assess the spectral match robustness of their method. They showed that their method is much less affected by experimental noise than the cosine score, which may suffer significantly in mass spectral library matching performance through the presence of even a single noise signal. While they showed that their method, alongside 26 alternative similarity scoring approaches, performed better than the cosine score, they did not include more recent machine learning-based similarity scores in their benchmarking study. If two MS/MS spectra of structurally similar or identical molecules (i.e., experimental and library MS/MS spectrum) are nearly identical, formula-based scoring methods that rely on mass fragmental overlap and heuristics such as the Entropy score can be expected to perform well (Li et al., 2021; Scheubert et al., 2017). However, structurally identical molecules can sometimes yield notably distinct MS/MS spectra if obtained with different instruments, different collision energies, or different analytical conditions. To address this issue, alternative approaches that can account for such differences have started to emerge.

For instance, Spec2Vec, an unsupervised machine learning model used to learn co-occurrence patterns of fragments and losses in spectral data, was successfully used for mass spectral library matching (Huber et al., 2021a). The approach first learns how to represent a MS/MS spectrum by an abstract feature vector, which is commonly referred to as embedding, or, in our specific case, a "mass spectral embedding". Typically, the goal of such approaches is to create an abstract feature space where similar elements (i.e., here MS/MS spectra) are close to each other. With Spec2Vec, for instance, the cosine score between two Spec2Vec-derived embeddings gives the spectral similarity score. The Spec2Vec’s mass spectral similarity score was evaluated with respect to its mass spectral library matching performance on a set of 95,320 MS/MS spectra from the GNPS libraries that comprised of at least 10 mass fragments after basic noise filtering. The authors showed that Spec2Vec’s scoring consistently outperformed cosine-based scores in accuracy over the full range of evaluated precursor tolerances in both retrieval rates and true/false positive rates. Another example of making use of a trained mass spectral embedding has been presented with MS2DeepScore (Huber et al., 2021b). This method aims to improve the scoring of structurally similar molecules based only on their obtained MS/MS spectra. It utilises a Siamese neural network that is trained on a large training set consisting of more than 100,000 MS/MS spectra of 15,000 molecules and has been evaluated on an independent dataset with 3600 spectra from 500 molecules. This Siamese network was trained with respect to the Tanimoto structural similarity of the training molecules using only their MS/MS spectra as input. While the model was not evaluated explicitly for mass spectral library matching purposes, the improved prediction accuracy of structural similarity scores achieved by this model (i.e., as compared to the modified cosine score) is a promising indicator for its mass spectral library matching potential.

Both machine learning-based and direct score-based approaches have their merits. Direct scores are easy to compute, conceptually simple to understand, and generally do not need any training data. However, they can be limited to simple heuristics and may fail to link spectra from molecules with more heavily differing fragmentation spectra between experimental platforms. Here, recent machine learning applications, like Spec2Vec and MS2Deepscore, provide promising alternatives to account for more complex fragmentation patterns to complement direct formula-based scores that rely on the mass fragmental overlap alone. *In-silico* fragmentation tools and their corresponding similarity functions provide another promising avenue for improving annotation rates through structural library matching. Given their technical nature, however, they are discussed in more detail in the machine learning for metabolite annotation section of this review.

### Analogue search

In addition to mass spectral matching for annotation and identification, querying and testing for chemical similarity of fragmented molecules is of great help during untargeted metabolomics experiments. There is a continuous development of new methods that allow for partial structural and spectral matching, also known as analogue search. Two rough strategies can be distinguished, i) those based directly on MS/MS spectra, and ii) those using machine learning for embedding-based scores or predictions.

In the former category, Hybrid Similarity Search (HSS), is a spectral pre-processing approach that augments MS/MS spectra to contain both the measured mass fragments and a single inferred neutral loss (Jang et al., 2019; Moorthy et al., 2017). Hybrid query spectra can then be matched with hybrid library spectra using conventional scoring approaches. The authors indicate that, for their method to work well, the query molecule needs to have a cognate molecule in the reference library with just a single structural difference that does not significantly affect fragmentation patterns (i.e., in general, having the same mass fragments with or without the structural difference). Despite this limitation, HSS finds structural similar molecules within the same chemical class in 85% of the queries (demonstrated by 4153 queries from 11 chemical classes in total with the NIST17 library). A generalisation of mass spectral matching for multiple neutral losses is available in the form of the Core Structure-based Search (CSS) algorithm (Xing et al., 2020). CSS calculates all possible neutral losses in the query and reference spectra and matches these for CSS score calculation. The authors show that their method outperforms MS-Finder and CSI:FingerID in the CASMI 2017 challenge and that the novel CSS method correlated better with a score for chemical similarity in comparison to other commonly used mass spectral similarity scores. Recently, Aisporna and co-authors introduced a large mass spectral library based on neutral losses alone and show how it connects structurally similar molecules using METLIN (Aisporna et al., 2022). In that work, however, no large-scale benchmarking was performed to show how it differs from cosine-based and modified cosine-based mass spectral comparisons.

Another alternative approach for structural matching based only on spectra is the SIMILE algorithm developed by Treen and colleagues (Treen et al., 2021). The method mimics DNA/protein sequence alignment to improve structural similarity measures for metabolomics research (Fig. 1c). First, a specific fragment ion substitution matrix is generated using all intra- and inter-MS/MS spectra differences of both to be compared spectra. Then, using dynamic programming, SIMILE finds the optimal path to match different fragment paths. The authors state that SIMILE finds ~ 90% novel structurally similar pairs compared to the modified cosine score (on the NIST2020 library filtered for [M-H] and CE between 5 and 40 eV).

The previously discussed Spec2Vec and MS2Deepscore methods are promising approaches to use for analogue searches for two main reasons. The first reason is that Spec2Vec and especially MS2Deepscore still predict similarity well for molecules that are not identical yet very similar due to the presence of several chemical modifications, while this is often not the case for methods directly based on MS/MS spectral similarity methods like the modified cosine score. The latter typically excel in recognizing very similar MS/MS spectra or MS/MS spectra derived from two molecules with one distinct chemical modification. The second reason is that the fast computation of Spec2Vec and MS2Deepscore makes them very suitable for analogue searches: i.e., when doing library matching a strict preselection on precursor m/z difference reduces the number of spectral comparisons that has to be made. However, in analogue search, such a strict filtering is not possible resulting in more spectral pair comparisons. The high speed and scalability of machine learning methods like Spec2Vec and MS2Deepscore enable mass spectral comparisons without any preselection on precursor *m/z*. Early performance results on using machine learning embeddings for matching structurally similar molecules are very promising. For instance, the machine learning-based analogue searching tool MS2Query, which builds on the advancements of MS2Deepscore and Spec2Vec, showed improved analogue search performance compared to using the modified cosine score (de Jonge et al., 2022). With more MS/MS spectral data becoming available, it can be expected that the methods improve and become even more reliable in a broad set of use cases. Currently, more work is needed to extend training data sets and to diversify and stratify test datasets to give users a clearer picture of the method’s reliability for their respective use cases. We anticipate that additional research efforts will be carried out to improve on partial matching using substructures inferred from machine learning tools such as MESSAR (Liu et al., 2020) or MS2LDA (van der Hooft et al., 2016), but also based on combinations of structure predictions and chemical compound class overlaps in the top-K predictions from tools such as SIRIUS (Dührkop et al., 2019). Ultimately, we anticipate that machine learning-based scores will be readily available for mass spectral library matching and analogue search and enrich classical and practical untargeted metabolomics annotations.

### MS/MS spectral organisation approaches

As an old adage goes: The *whole is more than the sum of its parts*. The same is true for MS/MS spectra in untargeted metabolomics. While it is difficult and cumbersome to individually annotate MS/MS spectra of detected metabolites in an untargeted metabolomics experiment, organising them into groups can substantially facilitate and enhance their annotation. To this end, measured MS/MS spectra obtained from a single experiment are investigated by comparing their MS/MS spectra, where spectral similarities serve as a proxy for structural similarity. Those with high similarity are put close to another or into the same groups, while loosely similar spectra or unrelated ones are placed further apart or are not linked to each other (Aron et al., 2020; Watrous et al., 2012). Subsequently, overview illustrations using either undirected graphs or mass spectral networks, dimensionality reduction, or dendrograms are generated. Thereby, the observed yet unstructured chemical space is organised into more manageable “groups”, often referred to as clusters or molecular/spectral families. These groupings can then be used to facilitate manual or automatic propagation of identifications or (partial) annotations of spectra, thereby providing valuable additional information for biological and chemical interpretation of the unknown molecules (da Silva et al., 2018). The three main approaches to group mass fragmentation spectra used in the field are i) graph/network-based representations of mass spectral similarities, ii) lower dimensional embeddings of the spectra or intermediate learned structures, and iii) clustering-based approaches.

In the first category, currently, the most popular approach is molecular networking available on the GNPS platform (Aron et al., 2020; Nothias et al., 2020). It comes in two flavours, namely i) mass spectral-based networking, now also referred to as “classical molecular networking” (Wang et al., 2016) and ii) feature-based networking (Nothias et al., 2020). Whilst the first approach uses MS/MS spectra and organises them regardless of the chromatographic information (i.e., chromatographic peaks), the second approach takes a chromatographic peak-centric approach by combining a quantitative peak list with qualitative MS/MS data. Here, isomers that may remain hidden in the first approach may be distinguished using chromatographic information such as retention time or ion mobility information. The resulting groups or molecular families are illustrated as undirected graphs, where nodes represent consensus MS/MS spectra or single molecules, and edges represent a high spectral similarity. Graphs can be visualised either on the webpage of GNPS or in Cytoscape (Kohl et al., 2011). The main scoring method to compare MS/MS spectra on GNPS is the modified cosine score, which typically allows for one neutral loss (i.e., corresponding to the precursor ion difference of the mass spectral pair) to be considered when testing the spectral similarity. Recently added, the user can also compute the molecular networks using Spec2Vec similarity scores. The authors of Spec2Vec demonstrated that with Spec2Vec more densely populated molecular networks can be generated. Additionally to molecular networking, the GNPS platform also hosts large spectral libraries for annotation and many different, related workflows (e.g., MASST (Wang et al., 2020), NAP (da Silva et al., 2018), NPClassyfire (Djoumbou Feunang et al., 2016), Qemistree (Tripathi et al., 2021), ReDu (Jarmusch et al., 2020), and MS2LDA (van der Hooft et al., 2016)), many of which can be conveniently started directly from generated molecular networks. Another possibility to generate mass spectral networks is available via the matchms package (Huber et al., 2020). It provides high-level access to spectral matching and scoring functionality for mass spectral data including the two ML approaches Spec2Vec and MS2DeepScore. Pairwise comparisons of all MS/MS spectra can be calculated and exported to Cytoscape for illustration as molecular networks. As this approach requires the user to implement the respective data processing routine in the python programming language rather than specify it via a graphical user interface, it allows easily customising it to each dataset as well as comparing different parameter settings and spectral similarity methods in a semi-automated fashion. Finally, the popular data pre-processing tool MS-DIAL also provides a means to generate feature-based molecular networks directly from raw data. It allows the straightforward exporting of its own annotated peak tables to Cytoscape for molecular network visualisation (Tsugawa et al., 2015).

Traditional molecular networks have spectra or features as their nodes, and edges largely based on rule-based similarity scores, though Spec2Vec scores are also finding use. When considering the novel machine learning-based mass spectral similarity scores, we envision that the edges could take on additional chemical information. Furthermore, edges based on predicted chemical classification or substructure overlap could also be integrated to steer the mass spectral network topology. Tools designed for partial spectral annotation such as MESSAR or MS2LDA seem especially promising in this respect. Early work in this direction was already done in MolNetEnhancer, where molecular network nodes are augmented using information from multiple tools, including MS2LDA substructure discovery, GNPS library matching, and *in-silico* structure annotation from various other tools (Ernst et al., 2019). In principle, similar information could be used to annotate edges to show which MS/MS spectra are considered adjacent for exploratory purposes.

Molecular networking is a highly popular, versatile, and insightful spectral organisation approach. However, the graphs can be highly dependent on the parameters used, and molecular families appearing as disconnected groups obfuscate the interfamily similarity. Indeed, a problem of molecular networks is that they do not retain a global view of the spectral similarity landscape. Alternative grouping approaches based on dimension reduction and machine learning embedding present complementary information unavailable in molecular networks. Thus, an alternative to molecular networking is to use binned MS/MS spectra or a machine learning embedding of spectra (e.g., derived via Spec2Vec or MS2DeepScore), and subsequently represent this space in two- or three-dimensional projections (e.g., PCA, t-SNE, UMAP). Depending on the dimensionality reduction method used, the distance between two spectra will be informative of their similarity, information that is not present in molecular networks. For example, the falcon tool (Bittremieux et al., 2021) and MS2DeepScore (Huber et al., 2021b) have been used to generate such illustrations from large numbers of MS/MS spectra. An interesting tool combining Molecular Networking with a low-dimensional embedding is MetGem (Olivon et al., 2018). The tool allows calculating both molecular networks and low dimensional embedding plots on MS/MS spectra directly, where the latter techniques preserve spectral similarities and thus provide a global view of the chemical space, while the former allows the very popular local similarity landscape exploration. In order to facilitate the switching between representations, MetGem offers a rich user interface that allows jumping from a node in one representation to the same node in the other representation. In addition, it also allows mapping meta-information directly into the molecular networks and subspace illustrations.

Moving beyond molecular networking and dimension reduction approaches, Qemistree provides an alternative means of grouping spectral data based on hierarchical clustering (Tripathi et al., 2021). Here, hierarchical clustering utilises predicted structural fingerprints obtained via SIRIUS (Dührkop et al., 2019), CSI:FingerID (Dührkop et al., 2015), and ZODIAC (Ludwig et al., 2020), with the latter approach showing improved MS/MS spectral-based elemental formula assignment performance for larger-in-size molecules (i.e., > 500 Da). CSI:FingerID uses molecular fingerprints obtained from the MS/MS spectra and is currently the best-performing tool for *in-silico* metabolite annotation. Moreover, as chemical fingerprints also remove the intermediate layer of MS/MS spectra or embeddings and thus work closer to the actual structure of the predicted molecules, it can be expected that the fingerprints correlate strongly with the chemical structure. Thus, the fingerprint-based Qemistree similarity scoring approach can be reasonably expected to improve the structural similarity assessment over commonly used cosine scores.

Molecular networking and other tools aimed at organising spectral data are immensely useful to untargeted metabolomics. This is also reflected by the number of papers mentioning the term ‘molecular networking’ in the previous years (2010: 26 papers; 2015: 192 papers, 2021: 1480 papers; search in May 2022 on https://scholar.google.com). Current research efforts focus on improving the concept of molecular networking on several ends (e.g., annotation propagation) with spectral matching being one of them for both partial and complete, as well as on machine learning embedding approaches that promise to improve the grouping of chemically related molecules. Combined approaches that provide a link between the local spectral connections provided by mass spectral networking with the global similarity structure views of lower dimensional embeddings seem especially fruitful future research avenues, as they promise to greatly increase the ease of untargeted metabolomics data analysis and at the same time facilitate an inroad to data-guided parameter setting for key networking thresholds that define possible connections between MS/MS spectra.

## Machine learning for metabolite annotation

In many fields, machine learning (ML), and in particular deep learning (DL), have radically changed how large datasets are handled. Although DL is technically a subfield of ML, it is generally referred to separately from “classical” ML. Applying ML and DL techniques in research is often considered a paradigm shift since it replaces heuristic (e.g., rule-based) data analyses with data-driven algorithms. These data-driven algorithms learn to achieve a specific task from available data (i.e., input features) by using an automatic optimization process which is called training. ML and DL comprise a large set of algorithms and approaches, many of which have become fairly standard for data analysis and are widely applied in metabolomics (Liebal et al., 2020). A key element in applying ML is the careful and usually manual selection and pre-processing of the features available for model training. In contrast, DL approaches are generally described as being more “expressive”, meaning that DL techniques can learn more complex relationships from the data and handle higher dimensional data. They do so by learning how to construct higher-order features input data to perform a certain regression or classification task optimally. As a consequence, DL techniques are employed in many areas of computer vision as well as in natural language processing (NLP) (Baraniuk et al., 2020). In the field of metabolomics, those approaches are still in a much earlier phase and have not yet been widely adapted (Liu et al., 2021; Pomyen et al., 2020; Sen et al., 2020).

In the last few years, however, a growing number of studies demonstrated the potential of such techniques to outperform conventional approaches in both annotation precision and the degree of automatization of metabolomic analyses. In principle, DL promises to mimic scientists’ decision-making more natively, making it possible to apply DL techniques for de novo structure elucidation and metabolite annotation, without relying on manually handcrafted features, which arguably lowers the human-derived bias of the model. Unfortunately, the respective model’s performance is limited by the richness and diversity of the data it has been trained on. Presumably, humankind has only mapped a small proportion of the vast metabolic space that exists on earth, making ML and DL models inherently limited to the chemical space that is already known as well as making its generalisation to other, currently unknown metabolites challenging. As a result, there is a survivor bias at play when identifying potentially novel molecules, as novel molecules that are chemically similar to known molecules are more easily identifiable. Therefore, validating ML and DL models and inspecting their generalizability to unknown chemical spaces is a challenging but important field of research. Additionally, DL models’ decision-making is generally considered to be a black box. This makes getting insight into model decision-making cumbersome and, in some instances, even impossible.

When we look at fields like computer vision or natural language processing (NLP) we can start to draw analogies and project what ML and DL can likely achieve soon regarding metabolite annotation. Over the past few years, the available ML and DL toolsets have matured and now provide a rich repertoire of techniques suited for different tasks and data types. Several of the most impressive performance gains in computer vision and NLP were merely a combination of incremental improvements in computational approaches with largely improved datasets in terms of quantity and quality (Baraniuk et al., 2020). One example is the recent rise of transformer architectures in NLP (e.g., BERT and other BERT-like architectures), which was accomplished by huge datasets and larger model architectures (Wolf et al., 2019). Although DL approaches used in NLP are being successfully applied to mass spectral datasets, interest in applying different model architectures like graph neural networks seems to be lower despite their natural suitability for learning on molecular networks. Nevertheless, the most dominant limitation in applying DL in metabolite annotation now and in the near future is unlikely to be the available techniques but rather the amount and quality of available training data.

### Limited reference MS/MS data and strategies to cope with it

Publicly or commercially available reference mass spectral datasets include the MassBank (Horai et al., 2010), MassBank of North America (MoNA) (https://mona.fiehnlab.ucdavis.edu/), METLIN (Smith et al., 2005), NIST Mass Spectral Library (Phinney et al., 2013), Wiley GC–MS library, Golm Database (Hummel et al., 2007), Fiehn metabolomics database (Kind et al., 2009), mzCloud (https://www.mzcloud.org/), Human Metabolome Database (HMDB) (Wishart et al., 2022), and GNPS (Aron et al., 2020). The molecules these datasets describe overlap to varying degrees and some of these datasets include each other fully (e.g., GNPS includes HMBD) (Vinaixa et al., 2016). These datasets typically comprise a few 10-thousands of molecules, which are relatively small numbers considering the chemically possible molecular configurations even small numbers of atoms can form. For example, over a billion chemically feasible natural product isomers can be generated for the molecular formula C_10_H_15_O_5_ (McKay et al., 2021). It is of note that this number probably does not reflect the actual size of available biological chemical space. Nevertheless, well-annotated high-resolution MS/MS spectra will remain a precious resource in the nearby future.

Data augmentation is a very common and successful strategy to mitigate the problem of too little training data. In the original context of this term, data augmentation means creating more diverse training data by altering data points in ways which do not counteract the training purpose. A classic example is that images used to train DL models often undergo numerous transformations in a randomised manner, e.g., slight changes in the aspect ratio, cropping, or changes in brightness and noise. In the training of MS2DeepScore, data augmentation was used to slightly modify the input spectra (Huber et al., 2021b). In a wider sense, data augmentation could also mean the use of fully or partly synthetically generated data. In addition to making the available training data more diverse, this approach can also extend the coverage of the training set. This, of course, strongly relies on the quality of the generated data, i.e., how closely *in-silico* generated MS/MS spectra correspond to actual MS/MS spectra of the respective molecules. For instance, the usability of transformer-based DL architectures for doing mass spectral annotations was recently demonstrated with MassGenie (Shrivastava et al., 2021). To overcome the limitation of low amounts of metabolomics data, the authors of MassGenie used *in-silico* fragmentation to generate MS/MS spectra for about 6 million small molecules. Another example of data augmentation outside of spectrum generation is DarkChem, a DL model with a variational autoencoder (VAE) architecture that can predict chemical properties (e.g., drug-likeness, m/z, logP) and generate new molecules with similar properties (Colby et al., 2020). Such generative models can be used to build molecular structure libraries. A risk of these generative models is that the DL model is mostly trained on generated data and will hence only generalise well to actual data if the resemblance between generated and true data is high (enough).

A different strategy to cope with limited data is the use of transfer learning. Training of many DL models including transformers for specific tasks can generally be improved by pre-training (i.e., transfer learning) on related datasets, especially when target datasets are small or biased (Wolf et al., 2019). Pre-training can also be applied to computational metabolomics to improve automatic peak annotation (Gloaguen et al. 2020). A particularly interesting variant of this strategy might be the use of unsupervised methods (e.g., autoencoders) to pre-train networks on unlabeled data (i.e. no metabolite annotations linked to data), which is far more abundant than annotated data.

**Fig. 2:**
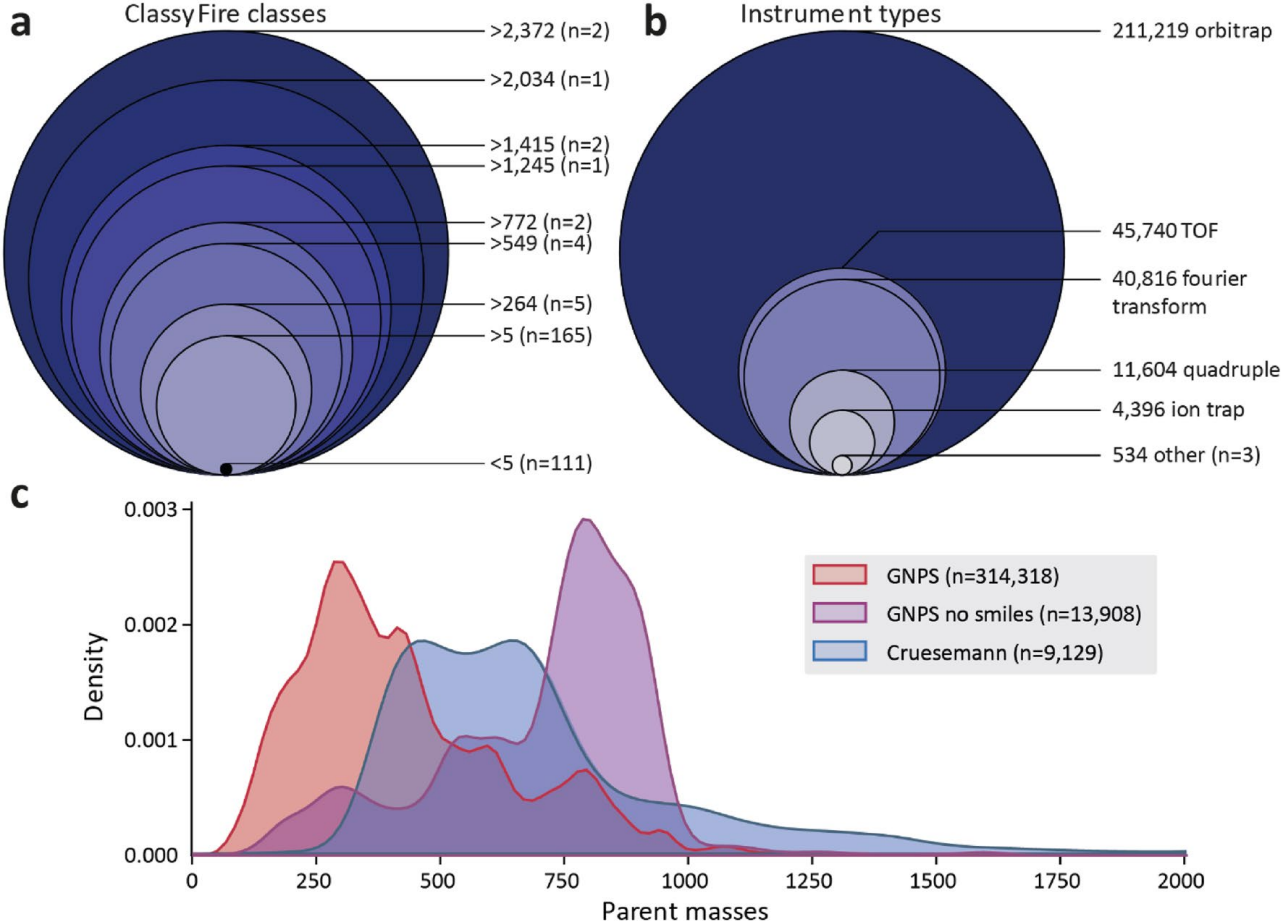
Illustration of reference library imbalances with respect to chemical classes, instrument types, and annotation rates by precursor mass. These factors may affect machine learning training dataset quality and representativeness. **a** ClassyFire classes of all 24,101 unique structures from the positive ionisation mode MS/MS spectra in GNPS. Chemical compound classes were determined by using ClassyFire superclasses (Djoumbou Feunang et al., 2016). For simplicity, classes are numbered from most to least occurring. **b** Instrument types for the 314,318 positive ionisation mode spectra in GNPS. Instrument type names were simplified to the ones shown in the figure. **c** Parent mass distributions of the 314,318 positive ionisation mode spectra in GNPS, the 13,908 positive ionisation mode spectra in GNPS that had no annotated SMILES, and the 9129 spectra in the dataset used by Crüsemann et al. (2015). Matchms was used to process the mgf files in the same way as in MS2DeepScore; here, MS/MS spectra with at least one fragment peak and a parent mass were considered

Another key limiting factor besides quantity and quality of available training data in ML/DL approaches are different sampling biases such as class imbalance (i.e., over- and under-represented classes in the training data). Compared to the quality and quantity of the training data, sampling biases are much harder to identify, and their adverse consequences are often very difficult to detect. Sampling biases are, despite thorough data preparation, easily inherited by subsequent machine learning models and can thus degrade the model’s performance to a certain degree. However, detecting sampling biases is a task of its own and typically requires extensively annotated training data, ideally also with additional information that is not primarily used for training and/or the prediction itself. Looking at the 24,101 structurally different metabolites (at the 2D-level, first 14 digits of their InchiKey) present in the GNPS library (accessed December 2021), the chemical compound classes ‘Prenol lipids’ and ‘Carboxylic acids and derivatives’ have the highest numbers of assigned molecules, while most classes are only assigned to a few metabolites, such as ‘Endocannabinoids’ and ‘Diazepanes’, which both are assigned to only 1 metabolite (Fig. 2a). Please note that chemical classification is somewhat subjective and dependent on the tool used. Nevertheless, we think that ClassyFire provides a good overview of what molecules are included in the GNPS library in general. The same is true for the representation of different instrument types and metabolite masses of MS/MS spectra. In the GNPS library, over 200,000 MS/MS spectra are measured with Orbitrap, while other instrument types such as qTOF and ion trap are much less common (Fig. 2b). In addition, parent masses of the MS/MS spectra in the GNPS library show a clear bias towards lower masses, with a peak around 300 Da (Fig. 2c). Comparing this distribution to an actual NP-rich dataset, like the 150 actinomycete strains analysed by Crüsemann et al., shows that mass spectral library distributions can be highly unrepresentative (Crüsemann et al., 2017). This could partially be explained by the fact that metabolites with higher masses tend to be harder to fully characterise, as is illustrated by the ~ 14000 MS/MS spectra in GNPS that do not have a fully resolved structure and that are mostly of higher molecular mass, such as lipids with unresolved double bond location (Fig. 2c).

Given this lack of representative metabolomics datasets for training, accurate de novo molecule annotation for more distant chemical entities is still not possible and such annotation workflows will remain reliant on expert curation for now. We expect that inferring molecule identity is only reliable when there is a high enough overlap with existing library entries. De novo metabolite identification by analytical chemistry experts implicitly includes more information about the sample than only the spectrum. Such information includes, for example, sample origin and chemical compound class. Future methods might choose to include such heterogeneous data to aim for increased model efficacy.

Strategies to overcome certain sampling biases are available, however, the respective bias and its extent must be known. One of the most basic approaches is over- and under-sampling, which means that data from under-represented classes will be used more frequently during training (and/or data from over-represented classes less frequently). This strategy was partly used in the training of MS2DeepScore by sampling training MS/MS spectra based on their InChIKey to avoid over-representation of molecules with high numbers of MS/MS spectra in the training data (Huber et al., 2021b). Another method to counteract class imbalance in training datasets is to weigh training samples unevenly. Still, strategies like over/under-sampling and differently weighing training samples are generally not able to fully circumvent adverse effects from severe sampling biases and are only a poor substitute for missing training data. Additionally, it is essential for the field that method developers clearly disclose the sampling biases and their extent in their data when they are aware of them.

### Different quality levels of spectra and how to deal with it

Another important factor in creating training data is to ensure adequate and consistent quality of mass spectral data. For instance, Li et al*.* note that their entropy scores display different distributions between NIST20, MassBank and GNPS (Li et al., 2021). Especially GNPS tends towards larger numbers of high entropy spectra due to higher spectral noise. These observed differences in entropy are caused by differences in experimental approaches, instrumentation and chemical matrices used, with GNPS contributions coming from a more diverse set of methodologies. For data curation, Li et al*.* suggest removing any signals with less than 1% of the base peak intensity. Applying this filter on GNPS data leads to entropy distributions more closely resembling those of MassBank. It is not uncommon for ML and DL training data to be subjected to data cleaning to improve the quality of the information on which future predictions will be based. It is important to share filtering settings used, as well as expectations with respect to data quality for the ML and DL tools, to ensure that their predictions are not disproportionately affected by high noise levels of experimental spectra, or by sequential applications of multiple filtering strategies.

### Strategies for machine learning driven metabolite annotation

**Fig. 3:**
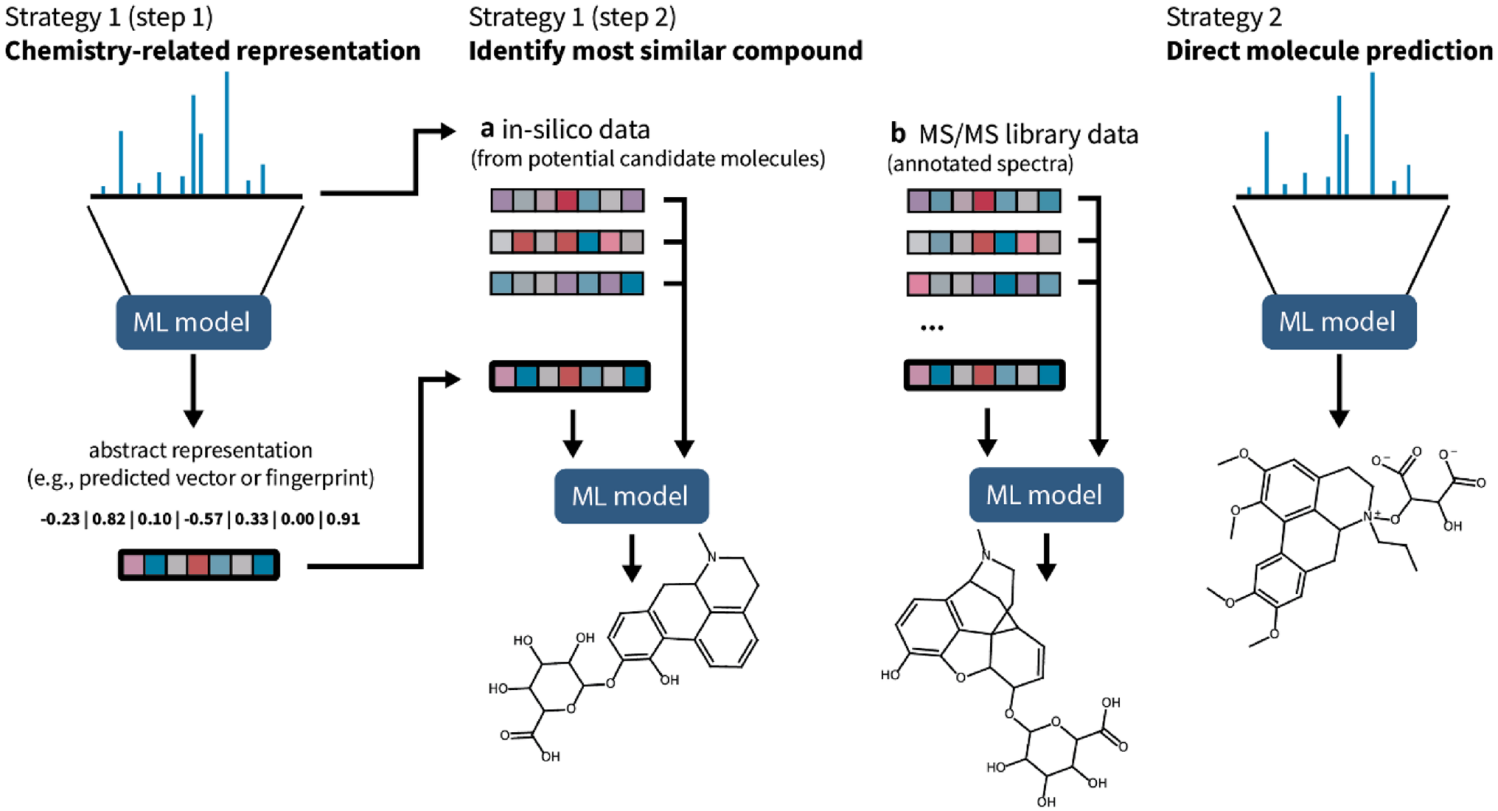
Two main machine learning (ML) based strategies applied today to link MS/MS spectra to molecules. Strategy 1 describes embedding-based library searches whereby chemically most related substances in a library are identified through comparisons of abstract embeddings of library molecules (step 1). This library can be expanded by including *in-silico* generated MS/MS spectra (step 2). Strategy 2 describes de novo structure elucidation directly from MS/MS spectra, circumventing any database comparison

Despite all limitations around current training data as described above, we observe that several different ML-based approaches already deliver very promising results for metabolite annotation. Here, we identify two different strategies (Fig. 3). These strategies have in common that they mainly rely on MS/MS data, i.e., fragment peaks and intensities. However, recently, additional and often complementary information such as instrument type or collision energy, retention time or order are also utilised for ML model training (Bach et al., 2022; García et al., 2022; Witting & Böcker, 2020).

The first strategy is generally not aimed at immediate metabolite annotation, but rather to translate MS/MS spectra into abstract representations that still are chemically meaningful although likely not understandable for anything but the trained model. DL models have been explored for directly predicting molecular fingerprints (Fan et al., 2020; Ji et al., 2020). Due to the under-representation of less common structural features in the training data, however, the focus was on predicting only frequently activated bits. An entirely different approach is Spec2Vec, which applies techniques from NLP to learn spectra representation in an unsupervised fashion (Huber et al., 2021a). The created representations are low-dimensional numerical vectors (embedding), which were shown to be able to find chemically related molecules. Using supervised training based on annotated spectra, MS2DeepScore is another DL approach that converts MS/MS spectra into abstract embeddings (Huber et al., 2021b). Similar to MS2DeepScore, Gleams uses a Siamese neural network to compare two MS/MS spectra and was trained on peptide spectra (Bittremieux et al., 2022). Strategy 1A relies on embedding-based library searches whereby chemically most related substances in a library are identified through comparisons of the abstract embeddings. In most cases, this will lead to identifying related rather than identical molecules since it -again- relies on the very limited coverage of the possible chemical space. To avoid the severe restrictions of the limited amount of reference standards, strategy 1B uses *in-silico* generation of spectra either as either the only source of reference data or in addition to existing reference standards. In most cases, large chemical databases such as PubChem are used to collect candidate molecules, e.g., by querying based on precursor mass. The chemical structure of those candidates is used to generate i*n-silico* spectra, which are then compared to the original query spectrum, typically using a wide range of different analysis pipelines which can include various ML and DL tools.

Examples of strategy 1 in combination with *in-silico* spectra matching are SIRIUS (Dührkop et al., 2019) and MetFID (Fan et al., 2020). Based on mass or formula, candidate structures are selected from a database and then compared to the query spectrum by comparing the reference molecular fingerprints to predicted fingerprints. The predicted fingerprints here are either computed from fragmentation trees (SIRIUS, using CSI:Finger ID (Dührkop et al., 2015)) or deep learning models (e.g., MetFID). Candidate selection, however, is not restricted to the use of molecular fingerprints and could in principle also be done based on chemically informed embeddings as provided by MS2DeepScore. This could be done, for example, by comparing distances between an unannotated embedding with annotated embeddings in a hyper-dimensional embedding space. COSMIC is an example of strategy 1 using *in-silico* data generation (Hoffmann et al., 2021), combined with a confidence scoring mechanism. COSMIC expands the known chemical space in a biologically inspired, semi-rule-based manner, in order to find more plausible candidate structures. Although COSMIC is clearly an improvement over previous models, accurately identifying false discoveries in metabolomics remains a challenge. In practice, the quality of the candidate selection relies on the quality of the predicted representations, but can also be improved by more elaborate selection algorithms such as Bayesian models (Dührkop et al., 2019) or other machine learning models (e.g., structured support vector machines in MetFID). Recently, LC-MS^2^Struct was proposed to integrate MS, MS/MS, as well as retention time information to increase the accuracy of the candidate structure selection (Bach et al., 2022).

In strategy 2 (Fig. 3), deep learning techniques are trained to directly predict chemical structures from MS/MS spectra. This concept is very much in line with the data-driven concepts behind applying DL models, which means that complex pipelines of many highly adjusted tools (as in strategy 1) could potentially be replaced by one model that learns to translate fragmentation patterns into chemical structures. Currently, however, this seems to be severely limited by the amount of available training data and its sparse coverage of chemical space. MassGenie is one of the first approaches to demonstrate what this might eventually look like. It uses a transformer architecture that is trained using 6 million *in-silico* generated spectra (Shrivastava et al., 2021). Not unexpectedly based on our previous considerations, it turns out that this model does not generalise well enough to be used for broad-scale structure prediction. Spec2Mol is another DL model for de novo structure prediction from mass spectral data using an encoder-decoder architecture GRU (Litsa et al., 2021). Interestingly, Spec2Mol can retrieve functional groups from spectra alone, but robust full structure elucidation is still a challenge for the model. Related transformer architectures were reported to work more reliably when restricted to the chemically more defined sub-space of peptides (Yilmaz et al., 2022). Using a combination of fingerprint and formula prediction with an encoder-decoder LSTM, MSNovelist (Stravs et al., 2021) can be seen as a hybrid between strategies 1 and 2, but mostly follows strategy 2 in avoiding the need for any comparison/candidate data. MSNovelist demonstrated that suitable deep learning models can already produce promising results and predict molecule structures for a notable fraction of the tested spectra. However, such approaches are still far away from replacing candidate or library matching approaches (Stravs et al., 2021). We note that for peptides it was demonstrated that DL can give very accurate predictions for retention time and fragment ion intensities (Gessulat et al., 2019). Regarding our prior discussion on the coverage of chemical space, however, it should be noted that peptides represent a very particular region of the chemical space with much higher-than-average coverage that can also be enriched *in-silico* in a more straightforward manner than generic small molecules. Peptides also have a much more straightforward fragmentation schema than other metabolites due to their modular properties.

### Perspective on machine learning for metabolite annotation

It is clear that MS/MS spectral-based small molecule structure elucidation remains a challenging task (Liu et al., 2021). The sheer size of the unexplored chemical space makes it practically impossible to create representative databases of experimental MS/MS data. Additionally, currently available datasets are skewed towards specific chemical classes. Moreover, different datasets exhibit different levels of noise. This implies that structure elucidation approaches based on similarity searches alone will be heavily impaired when investigating truly novel molecules. De novo structure elucidation from MS/MS data with ML and DL remains to be solved, although great initial strides have been taken. Initial methods focused on translating models from NLP to computational metabolomics, and more recent techniques from various other related fields are also beginning to make their appearance (e.g., transfer learning, adversarial methods, and graph-based models).

We would also like to emphasise that DL is not a surrogate for good scientific practices. Rich annotated data from well-performed experiments are paramount to develop an effective machine learning model. This also includes the use of community-adopted standard ontologies for those annotations (e.g., for naming mass spectrometry instruments or molecular structures). With the development of widely adopted standards already existing tools such as matchms can greatly help with combining MS/MS data from different sources (Huber et al., 2020).

As discussed, generative models have clear limitations and alone cannot be used to mitigate the issues relating to unrepresentative and biased datasets. State-of-the-art models that can link 50–70% of spectra to molecules accurately (or have a correctly predicted molecule in the top 10 of selected molecules) are not accurate enough to be used in practice. In order to improve structure elucidation in the short term, a hybrid DL and rule-based approach would be advisable (e.g., combining de novo structure elucidation with generated molecular libraries and fragmentation trees). At this moment, DL alone is not accurate enough to robustly infer molecule structure from MS/MS data, but by narrowing down the search field with for example sample meta-data, desired results become much more achievable. If we consider neighbouring fields such as proteomics, it is clear that with enough sufficiently varied training data, DL models should be able to learn biochemically relevant patterns from spectral data: i.e., with > 200,000 protein sequences with known 3D structure combinations, AlphaFold2 was able to make sequence-based 3D structure predictions of unprecedented quality (Jumper et al., 2021). Until sufficient metabolomics examples are available, combinations of rule-based and data-driven approaches are likely the most powerful road ahead.

## Benchmarking: test and training sets & good practice

With the development of many new computational tools, it is important that their performance is measured in a way that is objective and transparent and, ideally, allows a straightforward comparison to other tools. In metabolomics benchmarking we can separate two core components, i) the creation of a good test set of mass spectra, and ii) the metrics used to quantitatively evaluate different performance aspects of tools. The former determines how well benchmarking results are generalizable, while the adequate choice of the latter is critical for meaningful evaluations. However, there is no standard for benchmarking mass spectrometry-based metabolite annotation tools available currently, nor are there standardised test datasets. Here, we outline challenges and recommendations for the creation of meaningful and transparent tool evaluations and benchmarking studies.

### Selecting a good test set

It is generally accepted that test sets should reflect real data in order to provide accurate and realistic performance measures. Here, depending on the research context, we distinguish two benchmarking scenarios. The first approach aims to test the general applicability of a tool on a diverse set of use cases. These test sets should reflect the full diversity of chemical compound classes and mass ranges of real data. The second approach aims to show the performance for a specific use case, usually on which the tool performs particularly well. In-house created test sets would fall in the second category since these test sets are often not representative for all types of metabolomics experiments (i.e., metabolite types (i.e., chemical classes), mass ranges, instruments) and therefore the results cannot be expected to generalise well to all other use cases. Still, showing the relative tool performance on a custom test set can be valuable, since it can more clearly show what tool performs best in that concrete use case. However, it is important to clearly discuss the limitation of generalising these findings to the general chemical space.

When comparing tools to show their general applicability, doing a random selection of spectra from a large library may seem like a fair method that generalises well. However, this does not guarantee that the performance translates well to any real samples. Currently, only of a small percentage of all known metabolites there are annotated and authentic MS/MS spectra available (da Silva et al., 2015; Frainay et al., 2018). A glimpse of the low coverage of mass spectral libraries can already be caught through their much lower size compared to structural libraries. However, structural libraries themselves are not exhaustive either and hence the true scope of the dark matter of metabolomics is expected to be much larger still (da Silva et al., 2015). On top of this low coverage, it is expected that there will be sampling biases in the reference libraries, towards certain mass ranges, instrument types, and fragmentation parameters, amount of noise, chemical classes, organism-specific/model-organism metabolites or metabolomic pathways. For instance, Frainay et al. showed that some specific human metabolomic pathways are poorly covered by annotated MS/MS spectra (Frainay et al., 2018). Such sampling biases will often be a natural consequence of the way the data is generated, which also makes it very hard to avoid. Some spectra are easier to annotate, some compounds are more fragile or harder to measure, and some chemical classes are studied more extensively due to increased attention to certain research questions, sample types, or model organisms. Given the many reasons to expect strong biases as well as the obviously large discrepancies in chemical class representations in annotated reference mass spectral libraries (Fig. 2), we expect any test set composed of a randomly selected set of spectra from these libraries to inherit these biases. Therefore, such random test sets serve only as poor references for real application performance. The consequences of biased test sets become especially clear when benchmarking analogue search using mass spectral libraries. Analogue search test sets tend to inherit library biases such that the composition of test spectra is much more similar to the reference libraries than to the composition of actual real-world samples. This increased analogue density of test sets can easily lead to a substantial overestimation of method performance.

**Fig. 4:**
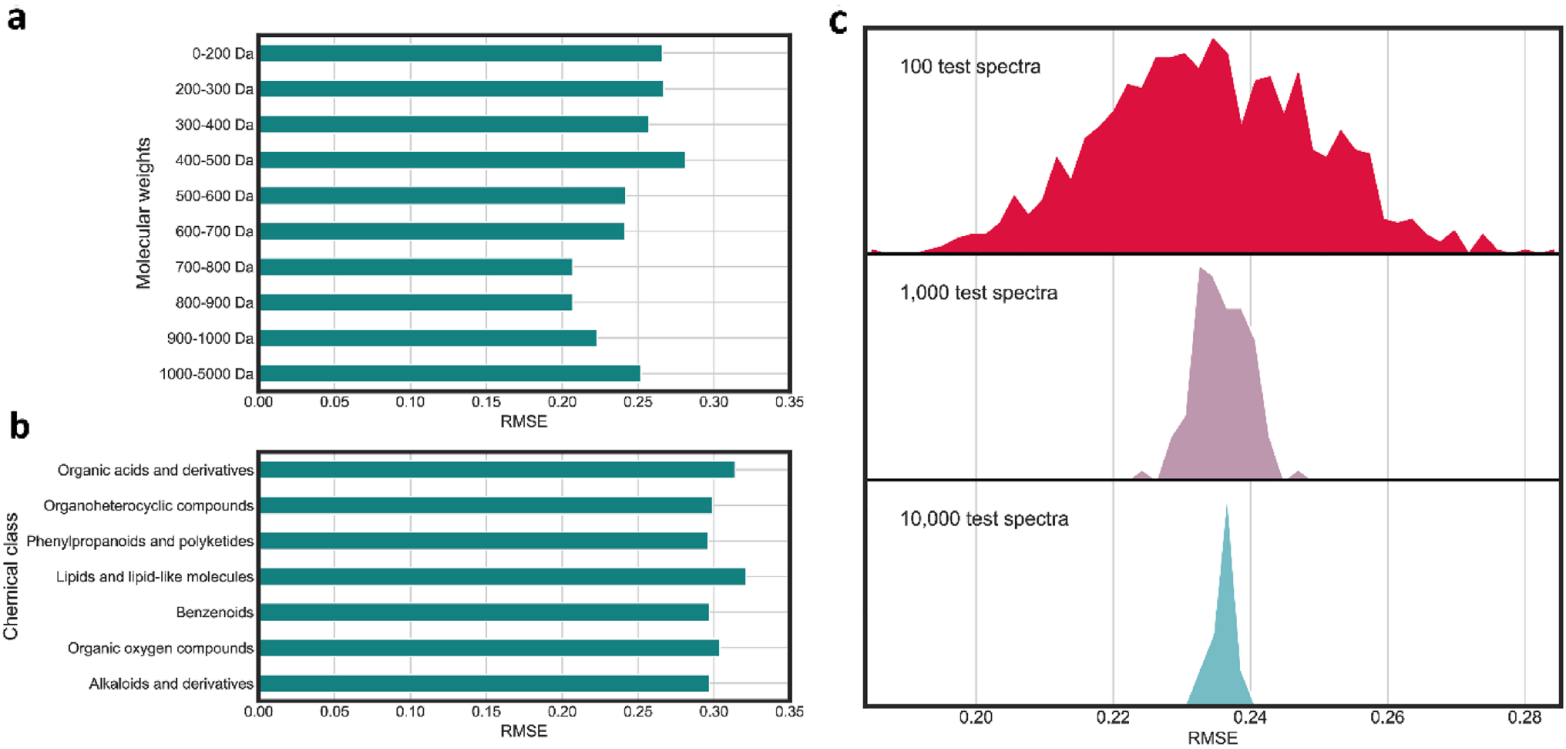
Benchmarking of MS2Deepscore with different types of test sets. In all figures the RMSE is determined separately for 10 Tanimoto score bins, followed by taking the average over these 10 bins. **a** RMSE of MS2Deepscore on test sets with 1500 spectra within a molecular mass range. **b** RMSE of MS2Deepscore on test sets with 1500 spectra of the most abundant ClassyFire superclasses. **c** Visualisation of the variance for different test set sizes. This shows there is a substantial difference between smaller test sets of 100 spectra

A method that can be used to correct for potential sampling biases in large libraries is using stratified sampling. Stratified sampling is an approach that ensures that the relative representation of the groups in your data represents real use cases. Examples of groups that can be used for stratification are chemical class or mass, taxonomic clades, utilised instruments and analytical methods and others. Stratification can be a good method to reduce sampling bias in your test set. However, it remains challenging to find a good way of stratification. Test datasets should be stratified such that they represent the use case scenario as closely as possible. The many highly different application domains of untargeted metabolomics make it impossible to create one unique stratified test set to represent this diversity of use cases. In the scenario of global tool comparisons, tool developers would like to evaluate their methods on a large and diverse set of spectra to indicate the wide applicability of their tools. Here, standardisation of the test set and good coverage of chemical space are essential to give insights into relative method performance. Stratification of the test sets improves this type of benchmarking in two important ways. On the one hand, stratification reduces the impact of arbitrary class imbalances on global performance metrics. On the other hand, stratification allows to make evaluations more concrete and transparent by allowing the inspection of these very same metrics on meaningful subsets of the test data, see Fig. 4 (e.g., chemical class, sample origin species or environments, weight category, instrument type). In the second benchmarking scenario, use case-specific performance evaluations may be done with small test sets stratified in a way to represent the particular use case scenario as closely as possible. While such specific evaluations are not expected to be generalizable beyond the target case, they provide specificity and unique data that global evaluations necessarily must glance over. As such, this second benchmarking scenario is expected to be performed by domain experts making use of their own reference standard sets, rather than by tool developers, and serves to complement the larger benchmarking studies.

The importance of selecting a test set that is a good representation of real use cases is critical since many tools have differences in performance for different chemical classes or mass ranges. Below we will discuss more concrete examples of the impact a test set can have on performance by illustrating the importance of molecular mass on the performance of the tools SIRIUS, Spec2Vec and MS2Deepscore.

Within SIRIUS, the number of possible fragmentation trees, and therefore the number of predicted molecular formulas that are computed increase exponentially with higher masses. This leads to reduced performance in molecular formula determination for masses higher than 500 Da (Böcker & Dührkop, 2016; Böcker et al., 2008). Using the recently developed ZODIAC method, this issue is partially resolved by reranking the lists of molecular formula candidates in larger MS/MS datasets leading to substantially lower error rates for molecular formula assignment (Ludwig et al., 2020). Besides this, fragmentation tree computation is an NP-hard problem and therefore puts a time constraint on the performance of SIRIUS. MS/MS spectra with masses above 850 Da are therefore currently not able to be computed within realistic timescales. To illustrate, the full Actinomycetes (*Salinispora/Streptomyces*) dataset used in MolNetEnhancer takes over 4 weeks to compute using the SIRIUS workflow, compared to around 24 h when using the same computational resources and a precursor mass cut-off of 850 Da. Test sets used for testing the performance of the different modules associated with SIRIUS consisted of very few spectra with higher masses. For example, spectra with masses above 700 Da were discarded when testing COSMIC, and the case studies in CANOPUS were restricted to spectra with masses below 860 Da (Dührkop et al., 2021; Hoffmann et al., 2021). In contrast, Spec2Vec was shown to perform less well when applied to spectra with lower (< 600 Da) masses. Selecting test sets with different mass ranges will therefore influence the performance of Spec2Vec, SIRIUS and other methods. This shows that selecting a balanced mass range is important in constructing a test set and should be reported transparently when a method is evaluated.

A good practice is to test the performance separately for specific mass ranges or chemical classes to illustrate if a tool has differences in performance. In Fig. 4 an example is given of how the performance for chemical classes (a) and mass ranges (b) could be tested and visualised in an example of MS2Deepscore. This also illustrates the impact molecular mass has on the performance of MS2Deepscore.

MS2Deepscore was benchmarked with test sets specific for certain mass ranges and chemical classes. All spectra in positive mode were selected from spectra from GNPS downloaded on 15–12–2021 and were cleaned using matchms (Huber et al., 2020). A very large test set was selected of 100,000 randomly selected spectra. This testset was used to create different subsets. From the remaining spectra, 204,318 were used as a training set to train an MS2Deepscore model and 10,000 spectra were used as a validation set. For each test set, the RMSE error was determined by comparing the prediction with the real Tanimoto score between two molecules. The comparison was done between all spectra in the test set, except for comparing to itself. E.g., for a test set of 100 spectra, almost 10,000 comparisons are made. Most comparisons between two randomly selected spectra/molecules result in low Tanimoto scores since the chance that a molecule is similar is low. A good similarity score predicts well across the whole range. Therefore, the RMSE was determined separately for 10 Tanimoto score bins, followed by taking the average over these 10 bins. This results in an RMSE averaged over Tanimoto bins, which is more representative of the performance of the model. Test sets for specific mass ranges and specific chemical classes were created. To select spectra in specific mass ranges 1500 spectra were randomly selected from each mass range and used as a test set (Fig. 4a). Chemical compound classes were determined by using ClassyFire superclasses (Djoumbou Feunang et al., 2016). Chemical classes were selected that had more than 1500 test spectra in the 100,000 test spectra, for each of these chemical classes 1500 spectra were randomly selected (Fig. 4b). To create Fig. 4c, the 100,000 test spectra were split to create test sets of specific sizes. 1000 test sets of 100 spectra were created, 100 test sets of 1000 spectra were created and 10 test sets of 10,000 spectra were created.

In addition to test sets being adequate representations of the target chemical space, it is important that test sets are large enough for performance metrics to be statistically accurate. Figure 4c shows that for test sets of 100 spectra, there is a large variance between such different test sets. Using larger test sets clearly reduces this risk of randomness in your test sets. When comparing methods on small test sets, variation in method accuracy due to the test set may exceed the actual difference in method performance, possibly leading to spurious conclusions with respect to relative method performance. Therefore, we strongly recommend using larger test sets. In some cases, using larger test sets is not an option, this could for instance be the case when analysing the performance for a specific mass range for which limited reference data is available. In these cases, either cross-validation or bootstrap approaches may be used to evaluate the statistical properties of the performance of the tool. In k-fold cross-validation, a dataset is split into k parts, with each part serving once as a validation set and the remainder serving as a training set. Repeated model training and validation give an indicator method performance variability across the k random sets and correspondingly what effective performance differences can be considered substantial rather than due to test set sampling alone. If repeated model training is computationally prohibitive, the bootstrap provides an opportunity to study performance via resampling of the test set. Here, the bootstrap sample performance variability gives an estimate of the variability of performance using only a single test set (Hastie et al., 2009; Kuhn & Johnson, 2013).

Another important factor to consider while benchmarking is the robustness to noise of the tested method. Some MS/MS spectra deposited in mass spectral libraries are already cleaned and are thus considered to be of high quality, while for other libraries, like many in the GNPS platform, this is not done yet. As such, it is common practice to first do some filtering and cleaning to prioritize relevant mass signals over noise (de Jonge et al., 2022; Huber et al., 2021a, 2021b). We do note that for machine learning-based methods that are not noise robust, pre-cleaning of mass spectral libraries comes with the risk of overestimating the model’s performance. Thus, when testing your dataset, it is important to be aware that this data was often already cleaned and filtered. To ensure that a method also works well with uncleaned input spectra collected for biological samples, which are generally noisier, it is key that filtering steps used for library and test mass spectra are also consistently applied to the real input spectra—and that the settings that were used are reported in the study. Further studies into the effect of noise filtering and spectral pre-processing in general for large-scale mass spectral comparisons are required to come up with more concrete recommendations.

### Metrics for performance

Besides the variety in options for selecting a suitable test set, there is a wide variety of options for different metrics for evaluating the performance of a tool. Below we will discuss the most common methods and their advantages and disadvantages.

Methods like spectral library matching, searching molecular structure databases or de novo structure prediction all have a similar aim to best predict the molecule belonging to a spectrum. The most straightforward method for benchmarking such methods is doing a prediction for a set of test spectra and comparing them to their ground truth. Often only the highest scoring hit is selected, but an alternative approach that is often used is taking the top-n hits (5 or 10) into consideration (i.e., a correct annotation is obtained when the correct hit is among the first n returned results) (Böcker et al., 2008). For a tool that aims to just be used as a first step for annotation and heavily relies on manual validation, looking at the top 10 hits is a useful metric. However, a risk of these methods is that many users may just look at the top hit, making the performance for the top 10 hits less relevant. We, therefore, argue that analysing the top 1 hit is the most informative approach and suggest top-n performance to be recorded in a complementary fashion rather than as a replacement. A specific case where top-n performance is relevant is for tools aiming at predicting substructures, since multiple substructures predicted for one molecule can be correct, reporting the number of correct hits in the top-n hits is relevant, as done for instance in the evaluation of MESSAR (Liu et al., 2020).

To evaluate the quality of the predictions the results are often evaluated in a binary fashion: the hit is correct or wrong. Often used metrics for evaluation of performance are accuracy, true-positive-rate or false-positive-rate. This method is easy to visualise and interpret, however, a downside of this method is that molecules that are predicted slightly wrong (e.g., small side group at the wrong position) are punished equally as predictions that are completely wrong. Therefore, a binary evaluation method is unsuitable for tools that have a slightly different aim than predicting the molecule belonging to a spectrum, for instance, an analogue search or a structural similarity score. An evaluation metric that tries to tackle this limitation of binary classification is evaluating the performance by calculating the structural similarity between the predicted molecule and the true annotation. Common methods used for predicting structural similarity are the Tanimoto/Jaccard coefficient, computed from molecular fingerprints. However, there is no consensus about what structural similarity score is best (Huber et al., 2021b). The interested reader is referred to Safizadeh and colleagues for more structural similarity approaches (Safizadeh et al., 2021). When using structural similarity scores for assessing the performance of a library search method, the predicted structure can be compared to the real structure. The performance can be assessed by calculating the RMSE for all test spectra or by visualising the distribution of the Tanimoto scores using a histogram. Using a structural similarity score to evaluate the performance of a similarity score (e.g., cosine score, Spec2Vec, MS2Deepscore) becomes a regression problem between the structural similarity and the spectral similarity. Typical metrics for evaluation are R2 and root-mean-squared-error rates, quantile–quantile plots (QQ-plots), as well as visual depictions of the structural and spectral similarity scores and their co-distribution.

Another important step in evaluating tools is the trade-off between recall and accuracy. Many tools do not always return a result for a MS/MS spectrum, but only if the score exceeds a certain threshold (for instance within a mass accuracy of 0.1 Da or a cosine score > 0.6). Specifying appropriate thresholds is often not trivial, in general using stricter thresholds will result in a lower recall but a higher accuracy. This trade-off can be visualised with a precision-recall curve. To make a fair comparison between different tools it is important to consider both the accuracy and the recall.

There is a lot of variation in computational time for different methods. A long computational time or the need for a lot of computational power can be limiting for some applications and it is therefore important to discuss. In addition, the computation time of some computational tools is strongly dependent on the size of the molecule that is processed, in these cases, this is also important to discuss. It is of note that a long(er) computational time does not always need to be a hampering factor: if the results can subsequently be used for a long and thorough analysis or can be quickly queried afterwards without the need for lengthy retraining of a model, and as such, this could work for a viable metabolite annotation strategy.

### Lack of effective method comparisons

Currently, there is a lack of comparison studies between different approaches for metabolite annotation, which makes it difficult for users to select the most appropriate method for their data analysis. One of the reasons why such a comparison has not systematically been carried out yet is that the different tools have a wide variety of goals such as spectral clustering, library or analogue searching, similarity scores or search for substructures, but also different strengths and weaknesses, like annotating small molecules or annotating large molecules. Instead of comparing tools to find the best tool out there, the focus should be on showing the strength and weaknesses of each tool to make it easier for a user to select a tool that best suits their needs. It would be very valuable to have large-scale comparison/benchmarking studies that highlight the strengths and weaknesses of the different scores and methods from the different applicable use case scenarios.

In addition to a variety of use cases, there are a lot of different datasets that could be used for validation purposes, making straightforward comparisons of tools difficult. A notable endeavour to harmonise such a comparison and to allow the different methods to blindly and thus fairly compare against each other are the CASMI challenges (http://casmi-contest.org). In this contest, the organisers put together a test dataset composed of only MS/MS spectra. This test dataset is given to the participants (i.e., developers of different *in-silico* metabolite annotation tools) without them knowing about the true identities of the metabolites behind the respective MS/MS spectra. The authors then apply their tools independently and the results are centrally compared with their respective ground-truth by the organisers of the CASMI challenge. We appreciate that studies use publicly available datasets for benchmarking, and we believe that this will be essential for effective performance comparisons showing the strengths and weaknesses of methods (as these can be reused without restrictions or financial requirements); however, the oftentimes small set of molecules used (i.e., few tens or hundreds), is unlikely to be sufficient to generalise to the many use cases of untargeted metabolomics. Nevertheless, the addition of new CASMI challenge MS/MS spectra to the public domain is always very much appreciated and very useful indeed.

### Conclusions benchmarking

Currently, there is no golden standard for compiling a good test set and how to evaluate a method's performance on it. Both the test set and the utilised metric will depend on the goals of a tool. However, since the selection of a test set and the metric can have a large impact on performance it is crucial to clearly discuss the limitations and biases of the selected test sets or metrics in publications. Besides a clear discussion of the limitations, there is a need for in-depth comparison studies that compare available tools. Instead of aiming to prove which tool is best, it is more relevant to show the strengths and weaknesses of each tool, for instance by showing the difference in performance for different mass ranges and chemical classes. In the short term, we propose the development of stratification schemes to guide the creation of large, stratified test sets that are randomly selected from mass spectral reference libraries, and to transparently highlight method performance for the different chemical classes and mass ranges, as well as of the different analytical methods that produced the input data. By doing so, aggregate performance measures have a clearer interpretation, and group-specific metrics can be evaluated for maximal transparency and insight. In the long term, we envision the development of standardised test sets that include informative subdivisions for straightforward comparison across studies, as well as the development of tools with functionality that enable end users to quickly and with minimal effort validate and compare different tools using their own in-house reference libraries.

## Overall conclusions

As we argue above, *in-silico* metabolite annotation methods in combination with large reference databases have the potential to transform the outcome of untargeted metabolomics approaches into structural information, thereby allowing for much finer-grained biological interpretations. This transformation has gained much traction and accelerated in recent years with a constant stream of novel tools improving upon existing methods and tools. Most *in-silico* approaches highlighted here can also be transferred to and used in many laboratories as a part of generic (open source) software thereby enabling large-scale applications of the computational methods. Moreover, with the publicly available spectral and chemical structure databases, which are continuously growing, also boosted by recent FAIR (i.e. Findable, Accessible, Interoperable, and Reusable) data sharing initiatives (Neumann, 2022), researchers and tool developers are starting to have uncomplicated and straight-forward access to structural and mass spectral data of myriads of molecules. This has fuelled the large-scale and repository-scale reuse of mass spectrometry data (Haug et al., 2019; Jarmusch et al., 2020, 2021; Sud et al., 2016), now also including retention time data (García et al., 2022). Furthermore, the adoption of standards such as the Universal Spectrum Identifier (USI) (Deutsch et al., 2021) will further aid in the harmonization of efforts, making access to large amounts of data for training and validation purposes much more straightforward.

Some of the most reliable methods to date employ machine learning and deep learning methods based on database similarity searching. However, the reach of these methods is limited to the covered chemical space in the used dataset for training. It has become clear that only a fraction of chemical space is covered, which makes it practically impossible to create representative databases of experimental MS/MS data. Training data generated from these databases will be biased towards certain chemical classes and mass ranges. This results in challenges for creating machine learning methods that generalize well to novel metabolites. Current efforts aimed at further homogenizing different datasets will help make sure that all available data can be used for training new ML and DL models and thus help increase their scope and performance. In addition, we suggest using stratification and over- and under-sampling to counteract biases in the data to be used in the short term. Since there is still a clear lack of (curated) metabolomics example data, combinations of rule-based and data-driven approaches are likely the most powerful. This would include developing and using robust *in-silico* MS/MS spectra generation in order to enrich our current datasets. Nonetheless, it remains crucial to increase the publicly available data and to focus on creating more reference data for underrepresented chemical classes. This is also important since de novo structure elucidation from MS/MS spectra remains a challenging task.

While DL developments in metabolomics are still in their infancy, there is reason to be optimistic about their future in the field. In the light of i) current advances in related fields that also look promising (i.e*.*, considering DeepDIA and DLEAMSE developed for proteomics (Qin et al., 2021; Yang et al., 2020), ii) the ever-increasing knowledge of how small molecules behave in the mass spectrometer (i.e., through quantum mechanics calculations (Lee et al., 2022), and iii) the increasing amount of training data, it is very likely that deep learning approaches will substantially boost the field. However, it is unlikely we will arrive there within the next 5—10 years. Until then, it will remain very important to make benchmarking possible, fair, and be explicit in what a method can, and cannot do, so that researchers can combine the right toolset for their task at hand.

We further note that the large number of novel tools makes it hard for users to judge which tool suits their needs best. While new tools are often benchmarked and compared with each other, there is a lack of standardized test data sets for critical performance evaluations and comparisons. This lack of standardization makes it difficult for end-users to find and utilize the most beneficial tools for their own experiments. A challenge in the standardization of benchmarking methods is that many tools developed have slightly different goals and therefore different benchmarking metrics are justified for different tools. Here, we discussed the pros and cons of often used benchmarking metrics and provide a set of recommendations to facilitate the understandable, fair, and reproducible benchmarking of metabolite annotation tools. We argue that, currently, the best approach is to use large, randomized test sets to show that a tool generalizes well, while also discussing the limitations due to potential biases. In addition, it is key to use specialized test sets to show the strength and weaknesses for specific subsets, like for instance specific mass ranges or chemical compound classes. This makes it possible for users to pick the best tool for their specific needs and focuses the field on improving the weaknesses of existing tools. More focus on sampling biases in data and in-depth benchmarking will remain key to preventing overestimation of the performance of tools. In addition to standards in benchmarking metrics, we believe the development of standard reference datasets to be crucial. Current efforts like CASMI are a great step in the direction of standardized benchmarking. However, we argue that in the future larger test sets should be used and to use subsets to benchmark the performance for specific chemical classes and mass ranges. We envision that if many groups support these recommendations, it will become easier to assess where, if, and how computational metabolomics tools are effective in adding biochemical information to metabolomics profiles.

Based on the currently ongoing community efforts, we expect that combined efforts in increasing the uniform coverage of publicly available data and the development of novel tools will rapidly improve the reliability of *in-silico* methods for untargeted metabolomics. Whilst the heterogeneity of the input data in metabolomics hampers progress in the field, we believe that with a concerted, harmonized, and community-based effort, metabolomics could also have its “AlphaFold moment” in the not too far distance.

## Data Availability

Software and data used to generate the figures of our study are available via https://github.com/vdhooftcompmet/hot-topic-computational-metabolomics.

## References

[CR1] Aisporna A, Benton HP, Chen A, Derks RJE, Galano JM, Giera M, Siuzdak G (2022). Neutral loss mass spectral data enhances molecular similarity analysis in METLIN. Journal of the American Society for Mass Spectrometry.

[CR2] Alseekh S, Aharoni A, Brotman Y, Contrepois K, D'Auria J, Ewald J, Fraser PD, Giavalisco P, Hall RD, Heinemann M, Link H, Luo J, Neumann S, Nielsen J, Perez de Souza L, Saito K, Sauer U, Schroeder FC, Schuster S (2021). Mass spectrometry-based metabolomics: a guide for annotation, quantification and best reporting practices. Natural Methods.

[CR3] Aron AT, Gentry EC, McPhail KL, Nothias L-F, Nothias-Esposito M, Bouslimani A, Petras D, Gauglitz JM, Sikora N, Vargas F, van der Hooft JJJ, Ernst M, Kang KB, Aceves CM, Caraballo-Rodríguez AM, Koester I, Weldon KC, Bertrand S, Roullier C (2020). Reproducible molecular networking of untargeted mass spectrometry data using GNPS. Nature Protocols.

[CR4] Bach, E., Schymanski, E. L., & Rousu, J. (2022) Joint structural annotation of small molecules using liquid chromatography retention order and tandem mass spectrometry data. *bioRxiv*.

[CR5] Baraniuk R, Donoho D, Gavish M (2020). The science of deep learning. Proceedings of the National Academy of Sciences USA.

[CR6] Beniddir MA, Kang KB, Genta-Jouve G, Huber F, Rogers S, van der Hooft JJJ (2021). Advances in decomposing complex metabolite mixtures using substructure- and network-based computational metabolomics approaches. Natural Products Reports.

[CR7] Bero SA, Muda AK, Choo YH, Muda NA, Pratama SF (2017). Similarity measure for molecular structure: A brief review. Journal of Physics: Conference Series.

[CR8] Bittremieux, W., Laukens, K., Noble, W. S., & Dorrestein, P. C. (2021) Large-scale tandem mass spectrum clustering using fast nearest neighbor searching. *Rapid Commununications of the Mass Spectrom.* 10.1002/rcm.9153 PMC8709870 34169593

[CR9] Bittremieux, W., May, D.H., Bilmes, J. and Noble, W.S. (2022) A learned embedding for efficient joint analysis of millions of mass spectra. *bioRxiv*. 10.1038/s41592-022-01496-1 PMC9189069 35637305

[CR10] Blaženović I, Kind T, Ji J, Fiehn O (2018). Software tools and approaches for compound identification of LC-MS/MS data in metabolomics. Metabolites.

[CR11] Blaženović I, Kind T, Torbašinović H, Obrenović S, Mehta SS, Tsugawa H, Wermuth T, Schauer N, Jahn M, Biedendieck R, Jahn D, Fiehn O (2017). Comprehensive comparison of in silico MS/MS fragmentation tools of the CASMI contest: Database boosting is needed to achieve 93% accuracy. Journal of Cheminformatics.

[CR12] Böcker S, Dührkop K (2016). Fragmentation trees reloaded. Journal of Cheminformatics.

[CR13] Böcker S, Letzel MC, Lipták Z, Pervukhin A (2008). SIRIUS: Decomposing isotope patterns for metabolite identification†. Bioinformatics.

[CR14] Colby SM, Nuñez JR, Hodas NO, Corley CD, Renslow RR (2020). Deep learning to generate in silico chemical property libraries and candidate molecules for small molecule identification in complex samples. Analytical Chemistry.

[CR15] Crüsemann M, O'Neill EC, Larson CB, Melnik AV, Floros DJ, da Silva RR, Jensen PR, Dorrestein PC, Moore BS (2017). Prioritizing natural product diversity in a collection of 146 bacterial strains based on growth and extraction protocols. Journal of Natural Products.

[CR16] da Silva RR, Dorrestein PC, Quinn RA (2015). Illuminating the dark matter in metabolomics. Proceedings of the National Academy of Sciences USA.

[CR17] da Silva RR, Wang M, Nothias L-F, van der Hooft JJJ, Caraballo-Rodríguez AM, Fox E, Balunas MJ, Klassen JL, Lopes NP, Dorrestein PC (2018). Propagating annotations of molecular networks using in silico fragmentation. PLoS Computational Biology.

[CR18] de Jonge NF, Louwen JR, Chekmeneva E, Camuzeaux S, Vermeir FJ, Jansen RS, Huber F, van der Hooft JJJ (2022). MS2Query: Reliable and scalable MS2 mass spectral-based analogue search. bioRxiv..

[CR19] Deutsch EW, Perez-Riverol Y, Carver J, Kawano S, Mendoza L, DenBossche TV, Gabriels R, Binz PA, Pullman B, Sun Z, Shofstahl J, Bittremieux W, Mak TD, Klein J, Zhu Y, Lam H, Vizcaíno JA, Bandeira N (2021). Universal Spectrum Identifier for mass spectra. Nature Methods.

[CR20] Djoumbou Feunang Y, Eisner R, Knox C, Chepelev L, Hastings J, Owen G, Fahy E, Steinbeck C, Subramanian S, Bolton E (2016). ClassyFire: Automated chemical classification with a comprehensive, computable taxonomy. Journal of Cheminformatics.

[CR21] Dührkop K, Fleischauer M, Ludwig M, Aksenov AA, Melnik AV, Meusel M, Dorrestein PC, Rousu J, Böcker S (2019). SIRIUS 4: A rapid tool for turning tandem mass spectra into metabolite structure information. Nature Methods.

[CR22] Dührkop K, Nothias L-F, Fleischauer M, Reher R, Ludwig M, Hoffmann MA, Petras D, Gerwick WH, Rousu J, Dorrestein PC, Böcker S (2021). Systematic classification of unknown metabolites using high-resolution fragmentation mass spectra. Nature Biotechnology.

[CR23] Dührkop K, Shen H, Meusel M, Rousu J, Böcker S (2015). Searching molecular structure databases with tandem mass spectra using CSI:FingerID. Proceedings of the National Academy of Sciences USA.

[CR24] Dunn WB, Erban A, Weber RJM, Creek DJ, Brown M, Breitling R, Hankemeier T, Goodacre R, Neumann S, Kopka J, Viant MR (2012). Mass appeal: Metabolite identification in mass spectrometry-focused untargeted metabolomics. Metabolomics.

[CR25] Ernst M, Kang KB, Caraballo-Rodríguez AM, Nothias L-F, Wandy J, Chen C, Wang M, Rogers S, Medema MH, Dorrestein PC, van der Hooft JJJ (2019). MolNetEnhancer: Enhanced molecular networks by integrating metabolome mining and annotation tools. Metabolites.

[CR26] Fan Z, Alley A, Ghaffari K, Ressom HW (2020). MetFID: Artificial neural network-based compound fingerprint prediction for metabolite annotation. Metabolomics.

[CR27] Fiehn O (2002). Metabolomics—The link between genotypes and phenotypes. Functional genomics.

[CR28] Frainay C, Schymanski EL, Neumann S, Merlet B, Salek RM, Jourdan F, Yanes O (2018). Mind the gap: Mapping mass spectral databases in genome-scale metabolic networks reveals poorly covered areas. Metabolites.

[CR29] García CA, Gil-de-la-Fuente A, Barbas C, Otero A (2022). Probabilistic metabolite annotation using retention time prediction and meta-learned projections. Journal of Cheminformatics.

[CR30] Gessulat S, Schmidt T, Zolg DP, Samaras P, Schnatbaum K, Zerweck J, Knaute T, Rechenberger J, Delanghe B, Huhmer A, Reimer U, Ehrlich H-C, Aiche S, Kuster B, Wilhelm M (2019). Prosit: Proteome-wide prediction of peptide tandem mass spectra by deep learning. Nature Methods.

[CR31] Hastie T, Tibshirani R, Friedman JH, Friedman JH (2009). The elements of statistical learning: Data mining, inference, and prediction.

[CR32] Haug, K., Cochrane, K., Nainala, V.C., Williams, M., Chang, J., Jayaseelan, K.V. and O’Donovan, C. (2019) MetaboLights: a resource evolving in response to the needs of its scientific community. *Nucleic Acids Research*. 10.1093/nar/gkz1019 PMC7145518 31691833

[CR33] Hoffmann MA, Nothias L-F, Ludwig M, Fleischauer M, Gentry EC, Witting M, Dorrestein PC, Dührkop K, Böcker S (2021). High-confidence structural annotation of metabolites absent from spectral libraries. Nature Biotechnology..

[CR35] Horai H, Arita M, Kanaya S, Nihei Y, Ikeda T, Suwa K, Ojima Y, Tanaka K, Tanaka S, Aoshima K (2010). MassBank: A public repository for sharing mass spectral data for life sciences. Journal of Mass Spectrometry.

[CR36] Huber F, Ridder L, Verhoeven S, Spaaks JH, Diblen F, Rogers S, van der Hooft JJJ (2021). Spec2Vec: Improved mass spectral similarity scoring through learning of structural relationships. PLoS Computational Biology.

[CR37] Huber F, van der Burg S, van der Hooft JJJ, Ridder L (2021). MS2DeepScore: A novel deep learning similarity measure to compare tandem mass spectra. J. Cheminform..

[CR38] Huber F, Verhoeven S, Meijer C, Spreeuw H, Castilla E, Geng C, van der Hooft J, Rogers S, Belloum A, Diblen F, Spaaks J (2020). Matchms—processing and similarity evaluation of mass spectrometry data. Journal of Open Source Software.

[CR39] Hummel J, Selbig J, Walther D, Kopka J (2007). The golm metabolome database: A database for GC-MS based metabolite profiling.

[CR40] Jang I, Lee J-U, Lee J-M, Kim BH, Moon B, Hong J, Oh HB (2019). LC–MS/MS software for screening unknown erectile dysfunction drugs and analogues: Artificial neural network classification, peak-count scoring, simple similarity search, and hybrid similarity search algorithms. Analytical Chemistry.

[CR41] Jarmusch AK, Wang M, Aceves CM, Advani RS, Aguirre S, Aksenov AA, Aleti G, Aron AT, Bauermeister A, Bolleddu S, Bouslimani A, Caraballo Rodriguez AM, Chaar R, Coras R, Elijah EO, Ernst M, Gauglitz JM, Gentry EC, Husband M (2020). ReDU: A framework to find and reanalyze public mass spectrometry data. Nature Methods.

[CR42] Jarmusch SA, van der Hooft JJJ, Dorrestein PC, Jarmusch AK (2021). Advancements in capturing and mining mass spectrometry data are transforming natural products research. Natural Products Reports.

[CR43] Ji H, Deng H, Lu H, Zhang Z (2020). Predicting a molecular fingerprint from an electron ionization mass spectrum with deep neural networks. Analytical Chemistry.

[CR44] Jumper J, Evans R, Pritzel A, Green T, Figurnov M, Ronneberger O, Tunyasuvunakool K, Bates R, Žídek A, Potapenko A, Bridgland A, Meyer C, Kohl SAA, Ballard AJ, Cowie A, Romera-Paredes B, Nikolov S, Jain R, Adler J (2021). Highly accurate protein structure prediction with AlphaFold. Nature.

[CR45] Kind T, Wohlgemuth G, Lee DY, Lu Y, Palazoglu M, Shahbaz S, Fiehn O (2009). FiehnLib: Mass spectral and retention index libraries for metabolomics based on quadrupole and time-of-flight gas chromatography/mass spectrometry. Analytical Chemistry.

[CR46] Kohl, M., Wiese, S., & Warscheid, B. (2011) Cytoscape: Software for visualization and analysis of biological networks. *Methods in Molecular Biology,* 291–303. 10.1007/978-1-60761-987-1_18 21063955

[CR47] Kuhn M, Johnson K (2013). Applied predictive modeling.

[CR48] Lee J, Kind T, Tantillo DJ, Wang L-P, Fiehn O (2022). Evaluating the accuracy of the QCEIMS approach for computational prediction of electron ionization mass spectra of purines and pyrimidines. Metabolites.

[CR49] Li Y, Kind T, Folz J, Vaniya A, Mehta SS, Fiehn O (2021). Spectral entropy outperforms MS/MS dot product similarity for small-molecule compound identification. Nature Methods.

[CR50] Liebal UW, Phan ANT, Sudhakar M, Raman K, Blank LM (2020). Machine learning applications for mass spectrometry-based metabolomics. Metabolites.

[CR51] Litsa, E., Chenthamarakshan, V., Das, P., & Kavraki, L. (2021) Spec2Mol: An end-to-end deep learning framework for translating MS/MS Spectra to de-novo molecules. *ChemRxiv*. 10.1038/s42004-023-00932-3 PMC10290119 37353554

[CR52] Liu, Y., De Vijlder, T., Bittremieux, W., Laukens, K., & Heyndrickx, W. (2021) Current and future deep learning algorithms for tandem mass spectrometry (MS/MS)-based small molecule structure elucidation. *Rapid Communication of Mass Spectrom,* e9120. 10.1002/rcm.9120 33955607

[CR53] Liu Y, Mrzic A, Meysman P, De Vijlder T, Romijn EP, Valkenborg D, Bittremieux W, Laukens K (2020). MESSAR: Automated recommendation of metabolite substructures from tandem mass spectra. PLoS ONE.

[CR54] Ludwig M, Nothias L-F, Dührkop K, Koester I, Fleischauer M, Hoffmann MA, Petras D, Vargas F, Morsy M, Aluwihare L, Dorrestein PC, Böcker S (2020). Database-independent molecular formula annotation using Gibbs sampling through ZODIAC. Nature Machine Intelligence.

[CR55] McKay, B. D., Yirik, M. A., & Steinbeck, C. (2021) Surge—A fast open-source chemical graph generator. *ChemRxiv*. 10.1186/s13321-022-00604-9 PMC9034616 35461261

[CR56] Members MSIB, Sansone S-A, Fan T, Goodacre R, Griffin JL, Hardy NW, Kaddurah-Daouk R, Kristal BS, Lindon J, Mendes P, Morrison N, Nikolau B, Robertson D, Sumner LW, Taylor C, van der Werf M, van Ommen B, Fiehn O (2007). The metabolomics standards initiative. Nature Biotechnology.

[CR57] Misra BB (2021). New software tools, databases, and resources in metabolomics: Updates from 2020. Metabolomics.

[CR58] Moorthy AS, Wallace WE, Kearsley AJ, Tchekhovskoi DV, Stein SE (2017). Combining fragment-ion and neutral-loss matching during mass spectral library searching: A new general purpose algorithm applicable to illicit drug identification. Analytical Chemistry.

[CR59] Neumann, J. (2022). FAIR data infrastructure. In S. Beutel, & F. Lenk (Eds.), *Smart biolabs of the future* (pp. 195–207). Springer International Publishing.

[CR60] Nothias L-F, Petras D, Schmid R, Dührkop K, Rainer J, Sarvepalli A, Protsyuk I, Ernst M, Tsugawa H, Fleischauer M, Aicheler F, Aksenov AA, Alka O, Allard P-M, Barsch A, Cachet X, Caraballo-Rodriguez AM, Da Silva RR, Dang T (2020). Feature-based molecular networking in the GNPS analysis environment. Nature Methods.

[CR61] Olivon F, Elie N, Grelier G, Roussi F, Litaudon M, Touboul D (2018). MetGem software for the generation of molecular networks based on the t-SNE algorithm. Analytical Chemistry.

[CR62] Peisl BYL, Schymanski EL, Wilmes P (2018). Dark matter in host-microbiome metabolomics: Tackling the unknowns–A review. Analytica Chimica Acta.

[CR63] Phinney KW, Ballihaut G, Bedner M, Benford BS, Camara JE, Christopher SJ, Davis WC, Dodder NG, Eppe G, Lang BE (2013). Development of a standard reference material for metabolomics research. Analytical Chemistry.

[CR64] Polishchuk, P.G., Madzhidov, T. I., & Varnek, A. (2013) Estimation of the size of drug-like chemical space based on GDB-17 data. *J. Comput. Aided Mol. Des.**27*. 10.1007/s10822-013-9672-4 23963658

[CR65] Pomyen, Y., Wanichthanarak, K., Poungsombat, P., Fahrmann, J., Grapov, D., & Khoomrung, S. (2020) Deep metabolome: Applications of deep learning in metabolomics. *Comput. Struct. Biotechnol. J.* **18**. 10.1016/j.csbj.2020.09.033 PMC7575644 33133423

[CR66] Qin C, Luo X, Deng C, Shu K, Zhu W, Griss J, Hermjakob H, Bai M, Perez-Riverol Y (2021). Deep learning embedder method and tool for mass spectra similarity search. Journal of Proteomics.

[CR67] Safizadeh H, Simpkins SW, Nelson J, Li SC, Piotrowski JS, Yoshimura M, Yashiroda Y, Hirano H, Osada H, Yoshida M (2021). Improving measures of chemical structural similarity using machine learning on chemical-genetic interactions. Journal of Chemical Information and Modeling.

[CR68] Scheubert K, Hufsky F, Petras D, Wang M, Nothias L-F, Dührkop K, Bandeira N, Dorrestein PC, Böcker S (2017). Significance estimation for large scale metabolomics annotations by spectral matching. Nature Communications.

[CR69] Schollée JE, Schymanski EL, Stravs MA, Gulde R, Thomaidis NS, Hollender J (2017). Similarity of high-resolution tandem mass spectrometry spectra of structurally related micropollutants and transformation products. Journal of the American Society for Mass Spectrometry.

[CR70] Sen P, Lamichhane S, Mathema VB, McGlinchey A, Dickens AM, Khoomrung S, Orešič M (2020). Deep learning meets metabolomics: A methodological perspective. Briefings in Bioinformatics.

[CR71] Shrivastava AD, Swainston N, Samanta S, Roberts I, Wright MM, Kell DB (2021). MassGenie: A transformer-based deep learning method for identifying small molecules from their mass spectra. Biomolecules.

[CR72] Smith CA, O'Maille G, Want EJ, Qin C, Trauger SA, Brandon TR, Custodio DE, Abagyan R, Siuzdak G (2005). METLIN: A metabolite mass spectral database. Therapeutic Drug Monitoring.

[CR73] Stein S (2012). Mass spectral reference libraries: An ever-expanding resource for chemical identification. Analytical Chemistry.

[CR74] Stravs MA, Dührkop K, Böcker S, Zamboni N (2021). MSNovelist: De novo structure generation from mass spectra. bioRxiv..

[CR75] Sud M, Fahy E, Cotter D, Azam K, Vadivelu I, Burant C, Edison A, Fiehn O, Higashi R, Nair KS, Sumner S, Subramaniam S (2016). Metabolomics Workbench: An international repository for metabolomics data and metadata, metabolite standards, protocols, tutorials and training, and analysis tools. Nucleic Acids Research.

[CR76] Sumner LW, Amberg A, Barrett D, Beale MH, Beger R, Daykin CA, Fan TWM, Fiehn O, Goodacre R, Griffin JL, Hankemeier T, Hardy N, Harnly J, Higashi R, Kopka J, Lane AN, Lindon JC, Marriott P, Nicholls AW (2007). Proposed minimum reporting standards for chemical analysis chemical analysis working group (CAWG) metabolomics standards initiative (MSI). Metabolomics.

[CR77] Treen DGC, Northen TR, Bowen BP (2021). SIMILE enables alignment of fragmentation mass spectra with statistical significance. bioRxiv..

[CR78] Tripathi A, Vázquez-Baeza Y, Gauglitz JM, Wang M, Dührkop K, Nothias-Esposito M, Acharya DD, Ernst M, van der Hooft JJJ, Zhu Q, McDonald D, Brejnrod AD, Gonzalez A, Handelsman J, Fleischauer M, Ludwig M, Böcker S, Nothias L-F, Knight R (2021). Chemically informed analyses of metabolomics mass spectrometry data with Qemistree. Nature Chemical Biology.

[CR79] Tsugawa H (2018). Advances in computational metabolomics and databases deepen the understanding of metabolisms. Current Opinion in Biotechnology.

[CR80] Tsugawa H, Cajka T, Kind T, Ma Y, Higgins B, Ikeda K, Kanazawa M, VanderGheynst J, Fiehn O, Arita M (2015). MS-DIAL: Data-independent MS/MS deconvolution for comprehensive metabolome analysis. Nature Methods.

[CR34] van der Hooft, J. J. J., Wandy, J., Barrett, M. P., Burgess, K. E., & Rogers, S. (2016) Topic modeling for untargeted substructure exploration in metabolomics. *Proceedings of the National. Academy of Sciences USA**113*. 10.1073/pnas.1608041113 PMC5137707 27856765

[CR81] Vinaixa M, Schymanski EL, Neumann S, Navarro M, Salek RM, Yanes O (2016). Mass spectral databases for LC/MS- and GC/MS-based metabolomics: State of the field and future prospects. Trends Analyt. Chem..

[CR82] Wang M, Carver JJ, Phelan VV, Sanchez LM, Garg N, Peng Y, Nguyen DD, Watrous J, Kapono CA, Luzzatto-Knaan T, Porto C, Bouslimani A, Melnik AV, Meehan MJ, Liu W-T, Crüsemann M, Boudreau PD, Esquenazi E, Sandoval-Calderón M (2016). Sharing and community curation of mass spectrometry data with global natural products social molecular networking. Nature Biotechnology.

[CR83] Wang M, Jarmusch AK, Vargas F, Aksenov AA, Gauglitz JM, Weldon K, Petras D, da Silva R, Quinn R, Melnik AV (2020). Mass spectrometry searches using MASST. Nature Biotechnology.

[CR84] Watrous J, Roach P, Alexandrov T, Heath BS, Yang JY, Kersten RD, van der Voort M, Pogliano K, Gross H, Raaijmakers JM, Moore BS, Laskin J, Bandeira N, Dorrestein PC (2012). Mass spectral molecular networking of living microbial colonies. Proceedings of the National Academy of Sciences USA.

[CR85] Wishart DS, Guo A, Oler E, Wang F, Anjum A, Peters H, Dizon R, Sayeeda Z, Tian S, Lee BL, Berjanskii M, Mah R, Yamamoto M, Jovel J, Torres-Calzada C, Hiebert-Giesbrecht M, Lui VW, Varshavi D, Varshavi D (2022). HMDB 5.0: The human metabolome database for 2022. Nucleic Acids Research.

[CR86] Witting M, Böcker S (2020). Current status of retention time prediction in metabolite identification. Journal of Separation Science.

[CR87] Wolf, T., Debut, L., Sanh, V., Chaumond, J., Delangue, C., Moi, A., Cistac, P., Rault, T., Louf, R., Funtowicz, M., Davison, J., Shleifer, S., von Platen, P., Ma, C., Jernite, Y., Plu, J., Xu, C., Scao, T.L., Gugger, S. et al. (2019). HuggingFace's Transformers: State-of-the-art Natural Language Processing.

[CR88] Xing S, Hu Y, Yin Z, Liu M, Tang X, Fang M, Huan T (2020). Retrieving and utilizing hypothetical neutral losses from tandem mass spectra for spectral similarity analysis and unknown metabolite annotation. Analytical Chemistry.

[CR89] Yang Y, Liu X, Shen C, Lin Y, Yang P, Qiao L (2020). In silico spectral libraries by deep learning facilitate data-independent acquisition proteomics. Nature Communications.

[CR90] Yilmaz M, Fondrie WE, Bittremieux W, Oh S, Noble WS (2022). De novo mass spectrometry peptide sequencing with a transformer model. BbioRxiv..

